# Supplementary material for: Healthy core: Harmonizing brain MRI for supporting multicenter migraine classification studies
Source: PLoS One. 2024 Dec 31;19(12):e0288300. doi: 10.1371/journal.pone.0288300 (PMC11687649; doi:10.1371/journal.pone.0288300)
Supplement: S1 Appendix — (DOCX) [file pone.0288300.s001.docx]

**Appendix**

*The proposed method, a unified framework of GFK+MMD*

Our proposed methodology leverages the Maximum Mean Discrepancy (MMD) and the Geodesic Flow Kernel (GFK) to harmonize neuroimaging data across different studies. This approach aims to mitigate dataset variability and improve the generalizability of classification models for migraine detection.

**Algorithm 1**: a proposed algorithm for Healthy Core

**Input:** Data $\mathbf{X}_{1}$ and Data $\mathbf{X}_{2}$;

**Output:** A single coreset $\{\mathbf{X}_{1c}, \mathbf{X}_{2c}\}$

**begin**

**repeat**

Iteration i =1 to $\mathbf{n}_{1}$ samples from $\mathbf{X}_{1c}$

Given $\mathbf{X}_{2c}$ Construct MMD matrix $\left\{ G(\mathbf{X}_{{1C}_{i}}),G(\mathbf{X}_{2C}) \right\}$

// $G$ is a positive semidefinite matrix, geodesic flow kernel (GFK)

Iteration j =1 to $\mathbf{n}_{2}$ samples from $\mathbf{X}_{2c}$

Given $\mathbf{X}_{1c}$ Construct MMD matrix $\left\{ G(\mathbf{X}_{1C}),G(\mathbf{X}_{{2C}_{j}}) \right\}$

// $G$ is a positive semidefinite matrix, geodesic flow kernel (GFK)

Compute rank by MMD and select sample to maximize decreasing the average MMD along $t$

**until** *Convergence (optimal point)*

Return

**Maximum Mean Discrepancy (MMD)**: At the core of our framework, MMD serves as a robust statistical measure to assess the similarity between two data distributions, P and Q, originating from separate datasets. It is defined as:

$MMD\left( \mathbb{P, Q ;}\mathcal{F} \right):=\sup_{f\mathcal{\in F}} \mathbb{| E}\left[ f\left( {DS}_{1} \right) \right]\mathbb{- E}\left[ f\left( {DS}_{2} \right) \right] |$ (1)

where $\mathcal{F}$ is a set containing all continuous functions [26]

where $\mathcal{F}$ represents a class of continuous functions. MMD effectively quantifies the distance between the distributions of two datasets by comparing their statistical properties across the entire feature space, rather than examining individual features independently [26].

**Geodesic Flow Kernel (GFK)**: Complementing MMD, the GFK approach utilizes the concept of Grassmann manifolds to generate meaningful new representations between two datasets. By exploiting geometric and statistical transformations across several feature representations, GFK facilitates the capture of data variability more comprehensively than single-representation methods. This process allows for the dynamic exploration of data properties and variations between datasets, thereby enhancing the adaptability and accuracy of cross-dataset analyses [43]. In our application of this unified GFK+MMD framework, it is crucial to note that MMD is applied to the entirety of the feature sets between DS1 and DS2, rather than conducting separate tests for each individual feature. This holistic approach ensures a more accurate and meaningful comparison of dataset characteristics, ultimately supporting the effective harmonization of neuroimaging data for improved migraine classification.

By integrating the strengths of both MMD and GFK, our method addresses the inherent challenges of multicenter neuroimaging studies, offering a robust solution for harmonizing datasets with varying properties and enhancing the performance of machine learning models in clinical applications.
