# Supplementary material for: Healthy core: Harmonizing brain MRI for supporting multicenter migraine classification studies
Source: PLoS One. 2024 Dec 31;19(12):e0288300. doi: 10.1371/journal.pone.0288300 (PMC11687649; doi:10.1371/journal.pone.0288300)
Supplement: Attachment — Submitted filename: PONE-D-23-16414_rev.pdf [file pone.0288300.s004.pdf]

PLOS ONE

Harmonizing Healthy Cohorts to Support Multicenter Studies on Migraine Classification using Brain MRI Data

--Manuscript Draft--

**Manuscript Number:**	PONE-D-23-16414
**Article Type:**	Research Article
**Full Title:**	Harmonizing Healthy Cohorts to Support Multicenter Studies on Migraine Classification using Brain MRI Data
**Short Title:**	Harmonizing Healthy Cohorts to Support Multicenter Studies on Migraine Classification using Brain MRI Data
**Corresponding Author:**	Teresa Wu<br>Arizona State University<br>Tempe, AZ UNITED STATES
**Keywords:**	migraine; magnetic resonance imaging; Classification; Cortical Thickness; Brain Structure; Headache; Cortical Surface Area; Brain Volume; Maximum Mean Discrepancy; Geodesic Flow Kernel
**Abstract:**	Multicenter and multi scanner imaging studies might be needed to provide sample sizes large enough for developing accurate predictive models. However, multicenter studies, which likely include confounding factors due to subtle differences in research participant characteristics, MRI scanners, and imaging acquisition protocols, might not yield generalizable machine learning models, that is, models developed using one dataset may not be applicable to a different dataset. The generalizability of classification models is key for multi-scanner and multicenter studies, and for providing reproducible results. This study developed a data harmonization strategy to identify healthy controls with similar (homogenous) characteristics from multicenter studies to validate the generalization of machine-learning techniques for classifying individual migraine patients and healthy controls using brain MRI data. The Maximum Mean Discrepancy (MMD) was used to compare the two datasets represented in Geodesic Flow Kernel (GFK) space, capturing the data variabilities for identifying a "healthy core". A set of homogeneous healthy controls can assist in overcoming some of the unwanted heterogeneity and allow for the development of classification models that have high accuracy when applied to new datasets. Extensive experimental results show that the utilization of a healthy core. One dataset consists of 120 individuals (66 with migraine and 54 healthy controls) and another dataset consists of 76 (34 with migraine and 42 healthy controls) individuals. A homogeneous dataset derived from a cohort of healthy controls improves the performance of classification models by about 25% accuracy improvements for both episodic and chronic migraineurs.
**Order of Authors:**	Hyunsoo Yoon, Ph.D.
	Todd J. Schwedt, MD
	Catherine D. Chong, Ph.D.
	Oyekanmi Olatunde, MS
	Teresa Wu, Ph.D.
**Additional Information:**	
**Question**	**Response**
**Financial Disclosure**<br><br>Enter a financial disclosure statement that describes the sources of funding for the work included in this submission. Review the submission guidelines for detailed requirements. View published research	YES,<br><br>This work was supported by the United States Department of Defense W81XWH-15-1-0286, Schwedt, Todd, (https://cdmrp.health.mil/search.aspx?LOG_NO=PR140037)

Powered by Editorial Manager® and ProduXion Manager® from Aries Systems Corporation

articles from *PLOS ONE* for specific examples.<br><br>This statement is required for submission and **will appear in the published article** if the submission is accepted. Please make sure it is accurate.<br><br>**Unfunded studies**<br>Enter: *The author(s) received no specific funding for this work.*<br><br>**Funded studies**<br>Enter a statement with the following details:<br>• Initials of the authors who received each award<br>• Grant numbers awarded to each author<br>• The full name of each funder<br>• URL of each funder website<br>• Did the sponsors or funders play any role in the study design, data collection and analysis, decision to publish, or preparation of the manuscript?<br>• **NO** - Include this sentence at the end of your statement: *The funders had no role in study design, data collection and analysis, decision to publish, or preparation of the manuscript.*<br>• **YES** - Specify the role(s) played.<br><br>* typeset	National Institutes of Health K23NS070891, Schwedt, Todd, (https://report.nih.gov/reportweb/web/displayreport?rid=568&ver=5)<br><br>and internal funds from the Mayo Clinic (Schwedt, Todd).
**Competing Interests**<br><br>Use the instructions below to enter a competing interest statement for this submission. On behalf of all authors, disclose any competing interests that could be perceived to bias this work—acknowledging all financial support and any other relevant financial or non-financial competing interests.<br><br>This statement is required for submission and **will appear in the published article** if the submission is accepted. Please make sure it is accurate and that any funding sources listed in your Funding Information later in the submission form are also declared in your Financial Disclosure statement.	The authors have declared that no competing interests exist.

View published research articles from *PLOS ONE* for specific examples.<br><br>**NO authors have competing interests**<br><br>Enter: *The authors have declared that no competing interests exist.*<br><br>**Authors with competing interests**<br><br>Enter competing interest details beginning with this statement:<br><br>*I have read the journal's policy and the authors of this manuscript have the following competing interests: [insert competing interests here]*<br><br>* typeset	
**Ethics Statement**<br><br>Enter an ethics statement for this submission. This statement is required if the study involved:<br><br>• Human participants<br>• Human specimens or tissue<br>• Vertebrate animals or cephalopods<br>• Vertebrate embryos or tissues<br>• Field research<br><br>Write "N/A" if the submission does not require an ethics statement.<br><br>General guidance is provided below. Consult the submission guidelines for detailed instructions. **Make sure that all information entered here is included in the Methods section of the manuscript.**	All studies were approved by the Mayo Clinic and Washington University School of Medicine in St. Louis Institutional Review Boards. All participants provided written consent prior to study participation. Individuals with migraine were identified from the headache clinics at both institutions.

Powered by Editorial Manager® and ProduXion Manager® from Aries Systems Corporation

**Format for specific study types**<br><br>**Human Subject Research (involving human participants and/or tissue)**<br>• Give the name of the institutional review board or ethics committee that approved the study<br>• Include the approval number and/or a statement indicating approval of this research<br>• Indicate the form of consent obtained (written/oral) or the reason that consent was not obtained (e.g. the data were analyzed anonymously)<br><br>**Animal Research (involving vertebrate animals, embryos or tissues)**<br>• Provide the name of the Institutional Animal Care and Use Committee (IACUC) or other relevant ethics board that reviewed the study protocol, and indicate whether they approved this research or granted a formal waiver of ethical approval<br>• Include an approval number if one was obtained<br>• If the study involved *non-human primates*, add *additional details* about animal welfare and steps taken to ameliorate suffering<br>• If anesthesia, euthanasia, or any kind of animal sacrifice is part of the study, include briefly which substances and/or methods were applied<br><br>**Field Research**<br><br>Include the following details if this study involves the collection of plant, animal, or other materials from a natural setting:<br>• Field permit number<br>• Name of the institution or relevant body that granted permission	
**Data Availability**<br><br>Authors are required to make all data underlying the findings described fully available, without restriction, and from the time of publication. PLOS allows rare exceptions to address legal and ethical concerns. See the PLOS Data Policy and FAQ for detailed information.	No - some restrictions will apply

Powered by Editorial Manager® and ProduXion Manager® from Aries Systems Corporation

A Data Availability Statement describing where the data can be found is required at submission. Your answers to this question constitute the Data Availability Statement and **will be published in the article**, if accepted.<br><br>**Important:** Stating 'data available on request from the author' is not sufficient. If your data are only available upon request, select 'No' for the first question and explain your exceptional situation in the text box.<br><br>Do the authors confirm that all data underlying the findings described in their manuscript are fully available without restriction?	
**Describe where the data may be found in full sentences. If you are copying our sample text, replace any instances of XXX with the appropriate details.**<br><br>• If the data are **held or will be held in a public repository**, include URLs, accession numbers or DOIs. If this information will only be available after acceptance, indicate this by ticking the box below. For example: *All XXX files are available from the XXX database (accession number(s) XXX, XXX.*).<br>• If the data are all contained **within the manuscript and/or Supporting Information files**, enter the following: *All relevant data are within the manuscript and its Supporting Information files.*<br>• If neither of these applies but you are able to provide **details of access elsewhere**, with or without limitations, please do so. For example:<br><br>*Data cannot be shared publicly because of [XXX]. Data are available from the XXX Institutional Data Access / Ethics Committee (contact via XXX) for researchers who meet the criteria for access to confidential data.*<br><br>*The data underlying the results presented in the study are available from (include the name of the third party*	Data from the study sponsored by the United States Department of Defense (DOD) will be made available through the Federal Interagency Traumatic Brain Injury Research (FITBIR) Informatics System in accordance with the rules and regulations of the DOD. Patient consent for the NIH-sponsored study and for the Mayo-funded study did not include a data sharing agreement.

Powered by Editorial Manager® and ProduXion Manager® from Aries Systems Corporation

and contact information or URL).<br>• This text is appropriate if the data are owned by a third party and authors do not have permission to share the data.<br><br>* typeset	
Additional data availability information:	

Powered by Editorial Manager® and ProduXion Manager® from Aries Systems Corporation

Manuscript

Click here to access/download;Manuscript;HealthyCORE_PLoS_One_Final.d

Harmonizing Healthy Cohorts to Support Multicenter Studies on Migraine Classification using Brain MRI Data

Hyunsoo Yoon[1], Todd J. Schwedt[2,3], Catherine D. Chong[2,3], Oyekanmi Olatunde[4], Teresa Wu[3,5]

[1] Yonsei University; Department of Industrial Engineering

[2] Mayo Clinic; Department of Neurology

[3] ASU-Mayo Center for Innovative Imaging

[4] Binghamton University; Department of Systems Science and Industrial Engineering

[5] Arizona State University; School of Computing and Augmented Intelligence

Corresponding Author:

Teresa Wu
Professor, School of Computing and Augmented Intelligence
Arizona State University
Adjunct Professor of Radiology, College of Medicine
Mayo Clinic
Founding Director, ASU-Mayo Center for Innovative Imaging
Teresa.Wu@asu.edu

Key Words: Migraine, Magnetic Resonance Imaging, Classification, Cortical Thickness, Brain Structure, Headache, Cortical Surface Area, Brain Volume, Maximum Mean Discrepancy, Geodesic Flow Kernel

Highlights

- The harmonization method was established by Healthy Core Construction.
- The inclusion of a healthy core addresses intrinsic heterogeneity that exists within a healthy control cohort and in multicenter studies.
- The utilization of a healthy core can increase the accuracy and generalizability of brain imaging-based classification models.
- The proposed harmonization method offers flexible utilities for multicenter studies.

Data Availability Statement

Data from the study sponsored by the United States Department of Defense (DOD) will be made available through the Federal Interagency Traumatic Brain Injury Research (FITBIR) Informatics System in accordance with the rules and regulations of the DOD. Patient consent for the NIH-sponsored study and for the Mayo-funded study did not include a data sharing agreement.

Abstract

Multicenter and multi-scanner imaging studies might be needed to provide sample sizes large enough for developing accurate predictive models. However, multicenter studies, which likely include confounding factors due to subtle differences in research participant characteristics, MRI scanners, and imaging acquisition protocols, might not yield generalizable machine learning models, that is, models developed using one dataset may not be applicable to a different dataset. The generalizability of classification models is key for multi-scanner and multicenter studies, and for providing reproducible results. This study developed a data harmonization strategy to identify healthy controls with similar (homogenous) characteristics from multicenter studies to validate the generalization of machine-learning techniques for classifying individual migraine patients and healthy controls using brain MRI data. The Maximum Mean Discrepancy (MMD) was used to compare the two datasets represented in Geodesic Flow Kernel (GFK) space, capturing the data variabilities for identifying a "healthy core". A set of homogeneous healthy controls can assist in overcoming some of the unwanted heterogeneity and allow for the development of classification models that have high accuracy when applied to new datasets. Extensive experimental results show the utilization of a healthy core. One dataset consists of 120 individuals (66 with migraine and 54 healthy controls) and another dataset consists of 76 (34 with migraine and 42 healthy controls) individuals. A homogeneous dataset derived from a cohort of healthy controls improves the performance of classification models by about 25% accuracy improvements for both episodic and chronic migraineurs.

1. Introduction

Neuroimaging studies have shown structural and functional alterations in brain regions involved in sensory-discriminative, affective, and cognitive pain processing, pain modulation, and multisensory integration in

should use ; instead of , to separate references ...

patients with migraine [Eck, J. et al. 2011, Lo Buono et al. 2017, Meylakh, N. et al. 2018, Mu, J. et al. 2020, Wei H. et al. 2020, Scheepens, D. S. 2020, Ashina, S. 2021]. Machine learning models trained to automatically classify individuals with migraine from healthy controls (HCs) using functional magnetic resonance imaging (fMRI) and structural MRI data have shown good utility for discriminating those with migraine from HCs. Yet, the generalization of the algorithms might be poor; that is, the classification model developed using one dataset may not perform as accurately on a separate dataset. The generalizability of classification models is key for multi-scanner and multicenter studies, and for providing reproducible results. Harmonization in clinical research has been focusing on defining, reviewing, and standardizing

uniformize: some publication years with parentheses others without

common data elements [Firnkorn, D et al. 2015, Yu, M. et al. (2018), Ma, D. et al. (2019), Chen, A. A. et

also, so authors inicials have . others do not

al. (2022)]. This is known as data curation - a strategy to combine the data from different sources to improve the generalizability of classification models. To be specific, this harmonization strategy is the explicit removal of the site or cohort-related effects in data from multiple sources. This harmonization strategy is desirable for improving the generalizability of classification models. Yet, from a data-driven aspect, the data from different sources may inherently exhibit different characteristics due to several reasons, such as differences in patient populations from which research participants are identified and the methods by which data are acquired. In machine learning research, extensive efforts are dedicated to transforming the data from different sources into a common feature space for evaluating similarities from multiple sources or adapting them thoroughly. This is also known as Domain Adaptation, a well-studied field [Tzeng, et al. 2017, Csurka et al. 2017, Tang, et al. 2020].

Domain adaptation leverages labeled data in one or more related source domains, to build a machine learning model for unseen or unlabeled data in a target domain. These adaptation methods aim to discover a shared feature representation for minimizing distribution differences while preserving the important

properties of original datasets. There are several types of domain adaptation approaches, such as re-weighting, parameter adaptation, feature augmentation, and feature transformation [Yao, Y et al. 2010, some authors do not have initials ... Gong, B 2012, Matasci, G 2015, Tzeng, et al. 2017, Csurka et al. 2017, Tang, et al. 2020]. After adaptation, a standard machine learning approach is used that assumes the test data are drawn from ~~a~~ similar distributions as the training data. As noted, most domain adaptation methods are designed to transform the data (both HCs and patients) together into a common space. Careful consideration of this approach reveals a potential concern, that is, it ignores the fact that there may exist heterogeneity even within the HC cohorts. As a result, transforming and aligning the whole dataset may not work as well as expected. In this research, we propose a new concept termed "healthy core": that transforms and aligns imaging data from HCs using their brain MRI data. Specifically, this study aims to 1) identify HCs from different sources to construct a healthy core; 2) develop and test classification models based on medical imaging that utilizes the healthy core in model training, validation, and testing across the datasets. We hypothesized that, compared to classification models developed without the use of a healthy core, imaging-based classification models for migraine developed with the use of a healthy core would have higher classification accuracy when applied to an unseen dataset.

2. Method

2.1. Approvals and Consent

All studies were approved by the Mayo Clinic and Washington University School of Medicine in St. Louis Institutional Review Boards. All participants provided written consent prior to study participation. Individuals with migraine were identified from the headache clinics at both institutions. HCs and individuals with migraine were enrolled from a database of research volunteers, and via community outreach.

2.2. Study Participants

Diagnoses of episodic migraine (EM) and chronic migraine (CM) were assigned using the most recent version of the International Classification of Headache Disorders available at the time of enrollment [ICHD-3 beta 2013]. Migraine diagnoses were assigned by a headache specialist (TS). HCs were excluded if they had a history of migraine, but occasional tension-type headaches (< 3 tension-type

why the different font size?

headaches per month) were allowed. Participants were male or female adults between the ages of 18-

years old

65. Those with contraindications to MRI, acute pain conditions other than migraine, neurological disorders

what about psychiatric disorders?

other than migraine, women who were pregnant, and individuals with abnormal brain MRI findings according to usual clinical interpretation were excluded. Participants were recruited for the study between 2012 and 2021.

2.3. Data Collection

Dataset 1 (DS1): The DS1 cohort, a total of 120 individuals (66 with migraine and 54 HCs) includes some patients scanned at Mayo Clinic Arizona and other patients scanned at Washington University School of Medicine in St. Louis.

extra space

Dataset 2 (DS2): The DS2 cohort, a total of 76 (34 with migraine and 42 HCs), consists of data from individuals scanned only at Mayo Clinic Arizona.

Data collected from all study participants included age, sex, Beck Depression Inventory-II (BDI-II) scores, and State-Trait Anxiety Inventory (STAI) scores; Additional data collected from migraine participants included headache frequency, number of years lived with headache, and Migraine Disability Assessment (MIDAS) scores.

Study participants were imaged on one of two Siemens (Erlangen, Germany) scanners, each at a different

all Siemens scanners have been called Magnetom something Skyra 20

institution. Scanners at both institutions differed in scanner model (Magnetom vs Trio), use of headcoil (12-

12

channel vs 20-channel), and T1-weighted and T2-weighted acquisition parameters, described in detail below.

Washington University: MAGNETOM Trio 3T scanner using a 12-channel head matrix coil

define acronyms

voxels are imaging volumes!

FOV is an area

T1-weighted images: TE=3.16 ms, TR=2.4 s, 1x1x1 mm^3 voxels, 256x256 mm^2 field-of-view (FOV), acquisition matrix 256 x 256. T2-weighted images: TE=88 ms, TR=6280 ms, 1x1x4 mm^3 voxels, 256x256 mm^2 field of view, acquisition matrix 256 x 256.

hyphenate field-of-view

what sequences were used? 2D or 3D? If 3D, MPRAGE for T1w, SPACE for T2? did you use T2w or T2 FLAIR weighted? From the parameters alone I would say you have used a 3D T1w sequence, namely MPRAGE. Both MPRAGE and FLAIR sequences use an inversion time TI! And what were the nutation/flip angles?

Mayo Clinic: MAGNETOM Skyra 3T scanner using a 20-channel head matrix coil.T1-weighted images: TE=3.03 ms; TR=2.4 s; 1x1x1.25 mm^3 voxels; 256x256 mm^2 field of view, acquisition matrix 256 x 256. T2-weighted images: TE=84 ms; TR=6800 ms; 1x1x4 mm^3 voxels; 256x256mm ^{2} FOV, acquisition matrix 256 x 256).

use a space between number and unit

remove parenthesis

Question: why the different parameters. This being said, some of the parameters, e.g TE, are not significantly different as far as image constrast is concerned!

T1 images were used for ruling out gross structural abnormalities.

Data included in this analysis have been utilized in prior analyses [Schwedt, T.J., 2015 and Chong, C. D. 2017]. T1-weighted image processing was performed using the automated FreeSurfer image analysis suite (version 5.3, http://surfer.nmr.mgh.harvard.edu/) available at the time of imaging. Image processing included skull stripping, automated Talairach transformation, segmentation of subcortical gray and white matter, intensity normalization, gray-white mater boundary tessellation, and surface deformation[Dale, A. M (1999), Fischl, B. (2001), Fischl, B. (2002), Ségonne, F. (2004)]. Data output measures from the automated ‘recon-all’ processing stream included regional cortical volume, cortical surface area, and cortical thickness over the left and right hemispheres.

If so, I would imagine that you have used 3D T1w MPRAGE sequences

space needed

2.4. Evaluation of Dataset differences between DS1 and DS2

For both datasets, measurements of cortical thickness, cortical surface area, and cortical volume for 68 regions were derived resulting in 204 measures per subject. Image features of HCs on cortical thickness, cortical surface area, and volumes in DS1 and DS2 were compared separately to identify the potential cohort difference. The comparison was performed for the means of features, the variances of features, and feature correlation structures. The cohort-wise means/variances of each feature were compared using a two-sample

you have not used subcortical volumes from the DSK atlas?

s

were variable distributions normal? if so state the testing and results. which significance level was used? 5%?

t-test/F-test. False Discovery Rate (FDR) was used to correct for multiple comparisons. The cohort-wise correlation matrices for the volume, area, and thickness features were compared using the two-sample Hotelling's T2 (the multivariate extension of the common two-group Student's t-test) and Multivariate Analysis of Covariance. In order to avoid the confounding of multiple cohort differences, the mean and variance differences were removed from each cohort to identify the remaining differences.

Did you use any particular software for conducting the statistical analysis?

2.5. Constructing the Healthy Core

Differences in imaging acquisition protocols, scanners, and even small differences in populations from which research participants are enrolled can lead to cohort effects. Therefore, simply combining all HCs from different studies/sites may not be optimal due to cohort effects. In order to address this issue, here we propose a new design strategy to discover the homogenous HCs from different datasets as referenceable HCs (see Step 2 in Figure 1). Our proposed approach contains two key concepts: 1) measure the similarity between HCs from different datasets, and 2) explore feature representations of the dataset to assess the similarities. First, the proposed method utilizes the Maximum Mean Discrepancy (MMD) [Gretton, A et al. 2012, Cui, P et al. 2020] which is a kernel-based statistic used to determine whether two distributions are similar. MMD can be considered as a criterion to determine whether subset samples from multiple datasets are homogenous [Gretton, A et al. 2012, Long, et al. 2015, Yan, et al. 2017, Epstein, et al. 2019, Zhang, et al. 2020, Kirchler, et al 2020]. However, the MMD is a point estimate-based statistic which may be too strict since the original datasets might inherently exhibit different characteristics. To capture the inherent variabilities, we hypothesize that Geodesic Flow Kernel (GFK), which utilizes geometric manifolds termed reference? Grassmann, may help. GFK has been widely used in the domain adaptation research field because it does not focus only on the feature representations of the two datasets (snapshot of each dataset), it constructs the transformation over the Grassmann space of the two datasets with a scale parameter. As a result, it has the potential to identify the subset samples from each dataset, for example, using MMD, as the homogenous core. ref?

I believe details are missing regarding the methods for constructing the healthy core. How did you do it in practice, how could the reader reproduce your approach?

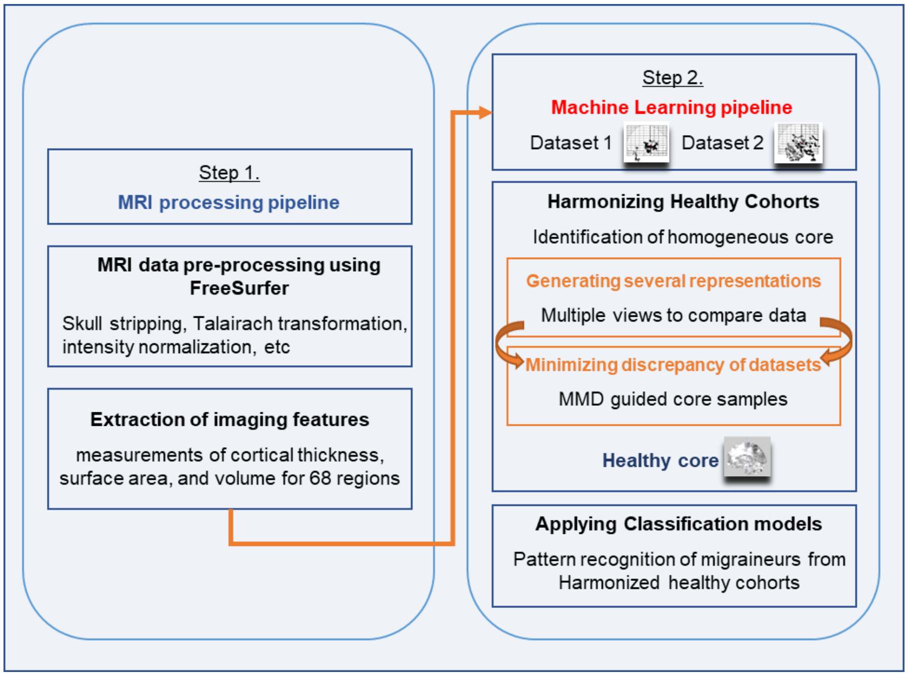

Figure 1. The overall workflow for Healthy Core Construction

We hypothesize that using a healthy core, a homogenous subset of HCs from different sources, can help improve the generalization of the machine learning model for classifying migraine since it addresses the heterogeneity of the HCs within and across the datasets; and sets the bridges between the datasets. To comprehensively assess the clinical utility of the proposed method, we designed three sets of experiments: (1) evaluating the performance of migraine classification models within a single dataset; (2) evaluating the performance of migraine classification models developed using one dataset and tested on a second dataset; and (3) evaluating the performance of migraine classification models with and without using healthy core samples for classifying individuals with CM and those with EM from a different dataset. In the training of the classification models, cross-validation has been applied. To get reliable results for the relatively small sample sizes, experiments are repeated under five different random scenarios (cross-validated) to build classifiers and report average performances. Since our last experiment focuses on migraine only, we report the Area Under the Curve (AUC) for the first two experiments and the accuracy of identifying migraine in the last experiment.

I would suggest including a subsection just for the classification tasks.

Need to provide more details on the classification tasks. sizes of training, validation and test sets.
were cross-validations done using randomly selected cases or every case was featured both in the training and validation set across folds? were training, validation and test sets balanced for age, sex, scanner parameters, pathology, etc? If so, what was the approach? was there any data augmentation? what were the algorithms used for classification?
Did you do hyperparameter fine-tuning? Any nesting?
You only used AUC and accuracy as figure of merits for the performance of your models?

3. Results

mean+-standard deviation, if so need to mentioned that

years old age range? 51+15?

DS1 consisted of 120 subjects (Age: 36±11, female:91, male:29, Amongst those with migraine, an average headache frequency was:8.91 ±6.25 days per month), including 54 HC, 51 EM patients, and 15 CM patients. DS2 consisted of 76 subjects (Age: 38±10, female:41, male:35, Average headache frequency for those with migraine: 17.97 ±8.08 days per month), including 42 HCs, 8 EM patients, and 26 CM patients. The overall sample numbers in this study are shown in Table 1 and the proportion of individuals with aura is described in Table 2.

space

were patient and HC groups age and sex matched?

is not one decimal place enough from the clinical point-of-view?

years old; age range? . The

one decimal place

Table 1. Overall sample numbers for HC, EM, and CM

	HC	EM	CM
DS1 (120 subjects)	54	51	15
DS2 (76 subjects)	42	8	26

should include the definition of acronyms either in the table caption or just below the table. Tables and figures should be on itself, and as reasonable as possible, self-understandable without resorting to the main text.

this is a parameter that was not mentioned in the methods section.

Table 2. Proportion of individuals with aura

	DS1 (66 migraineurs)		DS2 (34 migraineurs)	
	EM	CM	EM	CM
Aura	22	6	3	15
No Aura	29	8	5	11
Unknown	0	1	0	0

3.1. Dataset (Cohort) Differences

of which brain regions?

First, the mean of area, thickness, and volume features are significantly different between the HCs in the two datasets with a *p*-value<0.01 before the False Discovery Rate (FDR) correction. Further, three imaging

I would strongly suggest including supplementary data showing this.

which ones 41? and which 3?

features among those 41 significant features were identified as significantly different between two cohorts by the two-sample t-test and Kolmogorov-Smirnov test after FDR correction (Table 3).

this is not the difference, right? this shows the number of significantly different features ...

Table 3. The difference in the mean of features between DS1 and DS2

	Two-sample t-test	Two-sample Kolmogorov-Smirnov
Before FDR	41	43
After FDR	12	3

Figure 2 illustrates the significant mean difference in each imaging feature between the two cohorts. This result confirms that there are mean differences between the two cohorts for each modality.

need to show which features are these ...

too small lettering. cannot read properly.

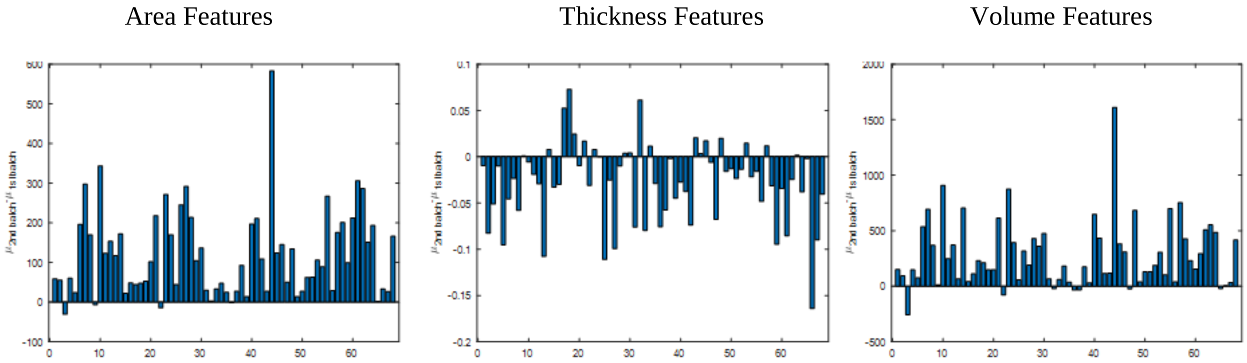

Figure 2. Univariate Analysis for Mean Difference Scale

Second, the variances of the features were tested. Table 4 shows that the variance for each feature is not significantly different between two cohorts under both tests after FDR correction.

Table 4. The difference in the variances of features between DS1 and DS2

	Two-sample *F*-test for equal variances	Ansari-Bradley Test for Equal Variances
Before FDR	8	9
After FDR	0	0

Furthermore, we investigated the possibility that significant group differences in sex distribution (*p*-value=0.01) contributed to the cohort difference.

this means that there are differences regarding sex, or that your significance level considered is 1%?

what does this mean in practice? how did you balance the samples?

We randomly selected the sex-balanced sub samples from both cohorts. After making the balanced groups,

define acronym.

the mean and variance of features (volume, area, and thickness) were tested using the t-test and KS test for mean, F-test and Ansari-Bradley test for variance whether the dataset difference exists. Since we selected

this should be stated in the methods section. did you do similarly for the remaining statistical tests?

a random subset to test, this test procedure was repeated 10,000 times to avoid a sampling-dependent conclusion. It was concluded that there is a significant mean difference and a marginal variance difference

for which brain region? are these overall values averaged for all brain regions? If so, why?

in each feature (volume, area, and thickness), but sex distribution was not a significant factor in the dataset difference.

Table 5 shows the average mean difference under several scenarios such as DS1 vs DS1, DS2 vs DS2,

you are accounting here both HCs and patients, right?

Random splits, and DS1 vs DS2. DS1 vs DS1 illustrates 21 randomly selected samples from the DS1 dataset and 21 non-overlapped samples from DS1. Note 21 samples are the number of consistent and balanced

what does this mean, balanced how?

samples across four scenarios. The random splits mean 21 randomly chosen subsets from the combined dataset (DS1 and DS2) and another non-overlapped 21 samples. The number in the table shows the mean

do not understand? where do you have the number of features?

features per each subset and their differences. Because of the randomness in the sampling, we repeat this task 100 times and report the averaged values.

what are the dispersion metrics of such differences ? (e.g. standard deviation)

Table 5. Average Mean Difference under Random Subsets within Dataset

Avg. |DS1-DS2|	DS1 vs DS1	DS2 vs DS2	Random splits	DS1 vs DS2
Area (mm^2)	82.12	77.86	80.75	102.27
Thickness (mm)	0.0416	0.0458	0.0436	0.0481
Volume (mm^3)	253.74	257.07	256.01	275.58

Table 5 confirms there is more of a difference between the two datasets (DS1 vs DS2) compared to sample subsets from within a dataset (e.g., DS1 vs DS1). The test results indicate that DS1 and DS2 are not

why do you say that? what is the criteria for compatibility?

compatible after mean and variance adjustments. Simple cohort-wise adjustments still have limited clinical applicability. This finding motivated us to develop new methods to construct a "healthy core" that consists of HC with similar characteristics from DS1 and DS2.

3.2. *Experiment I: evaluating the performance of different classification models within a single dataset*

The goal is to demonstrate the accuracy of machine learning models in classifying migraine within a single dataset as shown in table 6. In this experiment, we built four binary classifiers (regularized logistic regression, support vector machine (SVM), random forest, and XGBoost) to identify HC vs. CM, or HC vs EM. For each dataset (DS1 or DS2), we split the data into training (~60%), validation (~20%), and testing (~20%).

this information should come in the materials and methods section ...

T

why the choice of these ones?

with 5 folds, is that it?

but the folds include only the training and validation sets, right?

I suggest including a schematic in the materials and methods section about how you did the machine learning ...

Table 6. Single Dataset Experiments

use a more informative title.

	Dataset	Training	Validation	Test
CM	DS 1	HC (n=34) vs. CM (n=9)	HC (n=9) vs. CM (n=3)	HC (n=11) vs. CM (n=3)<br>accuracy chance level 11/(11+3) ~79%
	DS 2	HC (n=27) vs. CM (n=17)	HC (n=7) vs. CM (n=4)	HC (n=8) vs. CM (n=5)<br>accuracy chance level 11/(11+3) ~73%
EM	DS 1	HC (n=34) vs. EM (n=31)	HC (n=9) vs. EM (n=10)	HC (n=11) vs. EM (n=10)<br>accuracy chance level 11/(11+10) ~50%
	DS 2	HC (n=27) vs. EM (n=4)	HC (n=7) vs. EM (n=2)	HC (n=8) vs. EM (n=2)<br>accuracy chance level 8/(8+2) ~80%

you have quite small datasets.

again, always define the acronyms and abbreviations used in the table ...

Why did you consider only AUC as a model performance metric? This is not the best metric, especially in imbalanced datasets such as as yours. What are the chance values for the metrics?

what features were used for classification? all volumes, areas and thicknesses for all brain regions, or did you do any prior feature selection?

Table 7A. AUC of Four Classification Models in Single Dataset Experiments for CM

	Training on DS1	Test on DS1	Training on DS2	Test on DS2
Regularized Logistic Reg.	0.9600	0.7360	0.9820	0.6380
SVM	0.8720	0.6700	0.9800	0.5900
Random Forest	0.9320	0.7700	0.9180	0.6560
XGBoost	0.9480	0.8370	0.9040	0.7020
Average		0.7533		0.6465

maybe below chance level

below chance

are these mean values for the various folds? if so, please state that and show the dispersion as well.

Are performances significantly different across models?

Your results seem to show either overfitting or sample heterogeneity, or both...

Table 7B. AUC of Four Classification Models in Single Dataset Experiments for EM

	Training on DS1	Test on DS1	Training on DS2	Test on DS2
Regularized Logistic Reg.	0.8960	0.6660	0.8920	0.5760
SVM	0.9480	0.6740	0.9500	0.6060
Random Forest	0.9960	0.7400	0.9420	0.6280
XGBoost	0.8440	0.7060	0.9320	0.6440
Average		0.6965		0.6135

maybe above chance below chance

where are these algorithms coming from? did you coded yourselves? also, did you do any parameter optimization?

Four different models were applied to build the classifiers. The average AUC of the four models for HC vs CM was 0.7533 for the DS1 test dataset and 0.6465 for the DS2 test dataset as seen in Table 7A. The average AUC of the four models for HC vs EM was 0.6965 for the DS1 test dataset and 0.6135 for the DS2 test dataset as seen in Table 7B. We conclude that the machine learning models provide good performance during training, but that the performance deteriorates when using test data. This deterioration in accuracy is expected and the model performance may still be considered satisfactory.

why is it satisfactory?

e.g. the chance accuracy level for CM and DS1 is 11/(11+3) or ~79%. whilst this is not AUC this shows that your models should outperform this level. This being said, as a general rule of thumb, for 50% chance, any model and with AUC <0.700 is hardly useful for clinical use!

3.3. Experiment II: evaluating the performance of the classification models developed on one dataset and tested on a second dataset

The only model with performance above chance would seem to be DS1 for EM, with AUC values maybe nearing clinical relevance

Notice this model corresponds to the largest sample set and with balanced groups!

The goal is to assess the generalizability of machine learning models across datasets as shown in Table 8. Like Experiment I, here we tested on HC vs. EM, or HC vs. CM. Since one dataset can be fully utilized in training and validating with testing to be conducted in the second dataset, we split the data into training (~80%) and validation (~20%) for DS1, or DS2 respectively.

Table 8. Cross-Dataset Experiments

Datasets	Training	Validation	Test
CM<br>DS1> DS2	DS1:<br>HC (n=43) vs. CM (n=12)	DS1:<br>HC (n=11) vs. CM (n=3)	DS2:<br>HC (n=42) vs. CM (n=26)

chance level of 42/(42+26) ~62% accuracy

CM<br>DS2 > DS1	DS2:<br>HC (n=33) vs. CM (n=21)	DS2:<br>HC (n=9) vs. CM (n=5)	DS1:<br>HC (n=54) vs. CM (n=15)<br>chance level 54/(54+15) ~78%
EM<br>DS1 > DS2	DS1:<br>HC (n=43) vs. EM (n=41)	DS1:<br>HC (n=11) vs. EM (n=10)	DS2:<br>HC (n=42) vs. EM (n=8)<br>chance level 42/(42+8) ~84%
EM<br>DS2 > DS1	DS2:<br>HC (n=33) vs. EM (n=6)	DS2: include space<br>HC (n=9) vs.EM (n=2)	DS1:<br>HC (n=54) vs. EM (n=51)<br>chance level ~51.4%

Should also consider accuracy and other additional metrics

Table 9A. AUC of Four Classification Models for HC vs CM in Cross-Dataset Experiments

	Training (DS1)	Test (DS2)	Training (DS2)	Test (DS1)
Regularized Logistic Reg.	0.9160	0.5040	0.9340	0.6160
SVM	0.9740	0.580	0.8620	0.5980
Random Forest	0.9640	0.4400	0.9820	0.6900
XGBoost	0.9940	0.5740	0.8680	0.5880
Average		0.5245		0.6230

should be below chance level below chance level

Table 9B. AUC of Four Classification Models for HC vs EM in Cross-Dataset Experiments

	Training (DS1)	Test (DS2)	Training (DS2)	Test (DS1)
Regularized Logistic Reg.	0.8475	0.6375	0.9100	0.5900
SVM	0.7850	0.6050	0.9725	0.5825
Random Forest	0.9600	0.5325	0.9675	0.5950
XGBoost	0.8225	0.5375	0.9075	0.6175
Average		0.5781		0.5963

below chance maybe slightly above chance

As seen in Table 9A for HC vs CM, all four trained classifiers did not perform well in this cross-dataset experiment. The average AUC on models trained on DS1 dropped from 0.7533 (testing on DS1) to 0.5245 (testing on DS2), and the average AUC on models trained on DS2 dropped from 0.6465 (testing on DS2) to 0.6230 (testing on DS1). We can also observe similar patterns for HC vs EM in the cross-dataset experiment as seen in Table 9B. The average AUC on models trained on DS1 dropped from 0.6965 (testing on DS1) to 0.5781 (testing on DS2), and the average AUC on models trained on DS2 dropped from 0.6135 (testing on DS2) to 0.5963 (testing on DS1). This result demonstrates the existence of dataset discrepancies that need to be cautiously handled when building classification models.

I would say more relevant than that is the small sized datasets and the imbalanced groups

3.4.Experiment III: evaluating the performance of classification models with and without using Healthy Core

The goal is to assess the applicability of machine learning models developed in one dataset including participants with migraine and the healthy core to classify individuals with migraine from a separate dataset. The overall sample numbers in Cross-dataset Experiments with Healthy Core are shown in Table 10.

what is the composition of the healthy core? any particular subjects? any particular choice of features?

Table 10. Cross-dataset Experiments with Healthy Core

Datasets	Training	Validation	Test
CM DS1→ DS2	Healthy Core (n = 28) vs. CM from DS1 (n=12)	Healthy Core (n=28) vs. CM from DS1 (n=3)	CM from DS2 (n=26)
CM DS2→ DS1	Healthy Core (n=28) vs. CM from DS2 (n=21)	Healthy Core (n=28) vs. CM from DS2 (n=5)	CM from DS1 (n=15)
EM DS1 → DS2	Healthy Core (n = 28) vs. EM from DS1 (n=41)	Healthy Core (n=28) vs. EM from DS1 (n=10)	EM from DS2 (n=8)
EM DS2 → DS1	Healthy Core (n = 28) vs. EM from DS2 (n=6)	Healthy Core (n=28) vs. EM from DS2 (n=2)	EM from DS1 (n=51)

small test set

very small test set

You need to be much more detailed on the results regarding constructing the healthy core ...

are these values means across folds? if so please state that and the dispersion (e.g. standard deviation, std)

Table 11. Accuracy of four models with and without Healthy Core for CM and EM Classification

	Regularized LR		SVM		Random Forest		XGBoost	
	Without Healthy Core	With Healthy Core	Without Healthy Core	With Healthy Core	Without Healthy Core	With Healthy Core	Without Healthy Core	With Healthy core
Predicting CM from DS2	0.4900	0.6780	0.3790	0.7833	0.4700	0.7680	0.5650	0.8740
Predicting CM from DS1	0.6100	0.6920	0.4175	0.6325	0.4800	0.8325	0.5840	0.8400
Predicting EM from DS2	0.4716	0.7510	0.5550	0.8787	0.5950	0.8520	0.5520	0.8250
Predicting EM from DS1	0.4180	0.6250	0.4950	0.7400	0.5560	0.6600	0.4920	0.7920

define acronyms

Without using the healthy core, the four trained classification models do not have generalization accuracy to classify CM (test accuracy for DS2: 0.4760 – about 12 out of 26 CM, and test accuracy for DS1: 0.5229– about 8 out of 15) or EM (test accuracy for DS2: 0.5434 – about 4 out of 8 and test accuracy for DS1: 0.4903 – about 25 out of 51). Unlike typical approaches, the proposed method using the healthy core samples to build the same four classifiers achieved significantly better classification accuracy (see Table 11). These results show that the use of a subset of the HCs from different datasets has the potential to support the generalization of classification models.

are these average values across the different algorithms? if so please state that.
also consider including an extra column in table 11 with such mean+-std values

how many HCs from each dataset comprise the healthy core
what were the MMD values and any eventual GFK metric?

3.5. Discussions on Healthy Core

why not present the healthy core before the classification results?
in this manner you could follow a more consistent narrative, just as you present it in the material and methods section.

In Section 3.1, we conducted statistical analysis to confirm the dataset discrepancy between DS1 and DS2. For illustration, here we adopted t-Distributed Stochastic Neighbor Embedding (t-SNE), a technique for dimensionality reduction (e.g., based on principal component analysis) that is particularly well-suited for the visualization of high-dimensional datasets to demonstrate the impact of a healthy core. For details of t-SNE, interested readers are referred to [Van der Maaten 2008].

I would say fist and second dimensions for the different plots represent different things, right? actually they look the same for A and C and for B and D. Please discuss this.

why the dispersion of patient-related points, are these encompassing patient variability? would you expect and pattern also for patients?

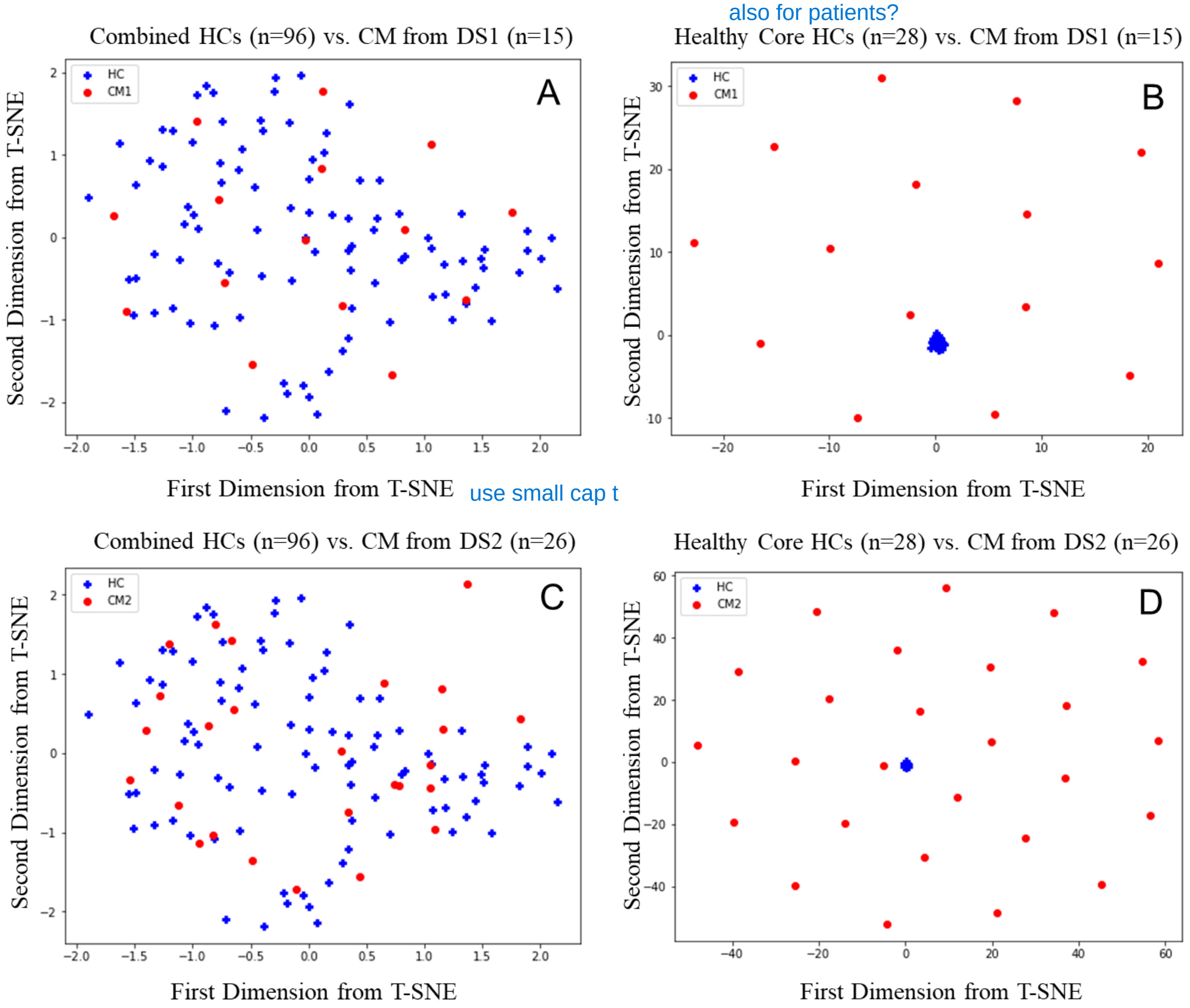

use small cap t

which HCs points correspond to DS1 and to DS2, is there any pattern? Maybe I would expect that given the differences you described in section 3.1

Figure 3. t-SNE plot of DS 1 and DS2 for CM on the first and second principal component. (A) combined HCs (n=96) vs. CM from DS1 (n=15). (B) Healthy Core (n=28) vs. CM from DS1 (n=15). (C) combined HCs (n=96) vs. CM from DS2 (n=26). (D) Healthy Core (n=28) vs. CM from DS2 (n=26).

As seen in Figure 3A and Figure 3C, combined HCs do not have separating (thus predictive) pattern compared to CM from either DS1 or DS2. On the other hand, the selected HCs cores have representative similar patterns, and healthy core (see Figure 3B and Figure 3D) can be well clustered in the t-SNE plot.

what encompasses the different dimensions? in particular the second dimension in B and D seems to be able to scatter patient-related points from a scale between -2 to 2 for A and C, to -50 to 60!

is why pattern from B so different from D and from B and D from fig. 3?

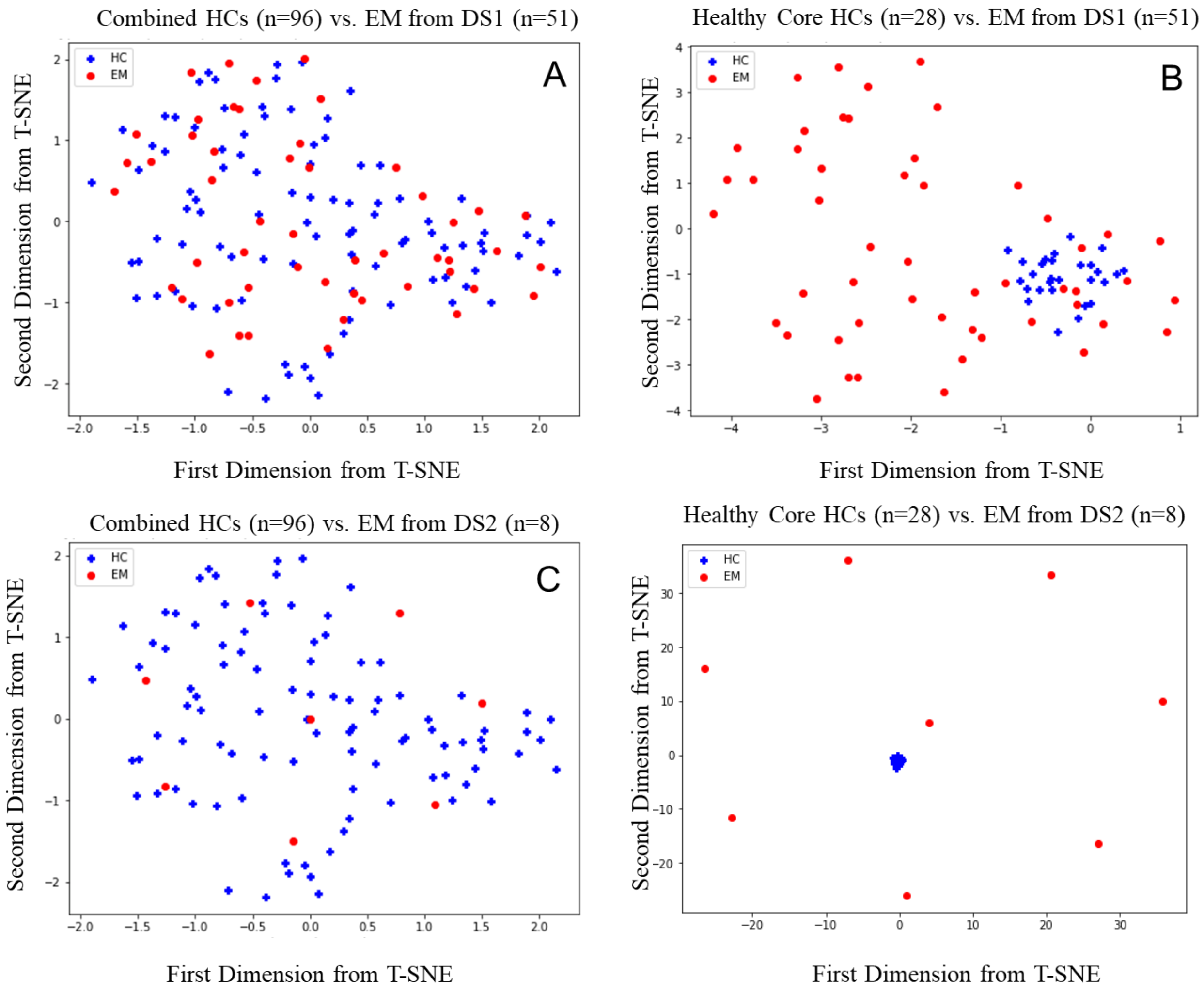

Figure 4. t-SNE plot of DS 1 and DS2 for EM on the first and second principal component. (A) combined HCs (n=96) vs. EM from DS1 (n=51). (B) healthy core (n=28) vs. EM from DS1 (n=51). (C) combined HCs (n=96) vs. EM from DS2 (n=8). (D) healthy core (n=28) vs. EM from DS2 (n=8).

Similar trends were observed for HC vs EM. Figure 4A demonstrated that the combined HCs contain mixed patterns with noise. However, 28 healthy cores identified by the proposed methods show compact representative patterns and are well separable from EM.

why do you call it noise?

are the features and dimensions the same for figures 3 and 4?

need to deepen the results presented, what are the main features that translate the HC group?
need to provide some ground basis for clinical/neurological/physiological interpretability ...

4. Discussion and Conclusion

The main finding of this study is that utilization of a healthy core, i.e. a homogeneous dataset derived from

CM and EM (present those in the order you show them along the manuscript)

a cohort of healthy controls, improves the performance of classification models for EM and CM. The use of a healthy core helps to overcome the deterioration in model performance that is otherwise seen when applying the migraine classification model to independent datasets collected from patients enrolled at different institutions and imaged on different scanners. Depending on the modeling approach, the inclusion of a healthy core resulted in classification accuracy as high as 88% for EM and CM.

The use of independent training and testing datasets is a substantial strength of the studies reported herein. Many prior publications (including some of our own) reported the accuracy of classification models for migraine train and test ~~the model~~ using the same dataset, with strategies such as the "leave-one-out"

include space

methodology[Schwedt, T.J. et al. 2015, Chong, C.D. et al., 2017, Lee M.J. et al., 2019, Zhang, Q. et al., 2018, Yang, H. et al., 2018, Chong, C.D., 2021]. Although this type of approach is valid and well-accepted,

, and therefore failing when it would be used in a completely ..

it likely leads to overestimating the performance of the model if it was used in a completely new and previously unseen dataset. Because independent training and testing datasets were used in the experiments described herein, the reported classification accuracies should be considered as more conservative estimates of model performance.

Maybe unnecessary statement or rephrase it ... it is not a more conservative estimate, not using independent dataset would result in hyped estimates ... maybe use considered a more robust

Useful classification models need to have high performance when tested on data that are collected from new patient populations and using collection techniques that might differ slightly from those used to collect the data that were included for model training (i.e. cross dataset accuracy). For example, a brain MRI-based classification model should still have high performance when tested on data from patients imaged at a different medical center and using different MR scanners. The experiments reported in this manuscript purposefully introduce this type of heterogeneity, with participants being enrolled from two different medical centers in two different regions of the United States. Results demonstrate the expected reduction in accuracy of the migraine classification models when they have been tested on independent datasets that include heterogeneity typical of multicenter imaging experiments. The experiments without the healthy

CM and EM

core that are reported herein demonstrate this: EM and CM classification accuracy was higher in the single

dataset experiments (CM accuracy 65%-75%, EM accuracy 61%-70%) compared to the cross-dataset experiments (CM accuracy 57%-59%, EM accuracy 58%-60%). The introduction of a healthy core helped to overcome this deteriorating model performance and provided much higher classification accuracies for
CM and EM
EM and CM.

When performing research that includes "healthy controls" there is an assumption that the healthy control cohort is homogeneous. However, even when stringent eligibility criteria are applied in an attempt to make the healthy control group as healthy as possible, there will always be identifiable and unidentifiable heterogeneity within the healthy control group due to differences in demographics, prior life experiences, physical and mental well-being, underlying genetic heterogeneity, the existence of diseases that have not yet manifest, and other sources of heterogeneity. When establishing "normal values" for certain types of tests, such as blood test results, for example, the use of very large sample sizes can mostly overcome this issue of heterogeneity within the healthy control cohort. However, available sample sizes are smaller for establishing normal values for brain MRI, a diagnostic test that is time and cost-intensive. The "healthy
need to describe much better. also, will the code be made available?
core" method described in this manuscript can help overcome this challenge. For example, with the use of the healthy core and XGBoost, the cross dataset classification accuracy improved to 84%-87% for CM and to 79%-83% for EM.

When using the healthy core, the classification accuracies for migraine are comparable, if not somewhat higher, than those reported previously in the literature. This is so even though independent training and testing sets were used. For example, prior studies using brain imaging structural data reported classification accuracies of 67%-86% for differentiating migraine from healthy controls [Schwedt, T.J. et al. 2015], while those using functional MRI data reported accuracies of 73%-86% [Chong, C. D. et al. 2017; Yang, H et al. 2018; Lee, M. J. et al. 2019]. Classification models including multimodality imaging data, combining structural and functional measures, have provided accuracies of 83%-84 [Zhang, Q. et al. 2016; Gaw, N. et al. 2018]. Classification accuracies for EM were generally lower than for CM in the experiments reported
. Prior
herein. This is consistent with expectations since CM is the more severe disease state, prior brain imaging

studies have demonstrated that headache frequency has a positive correlation with the amplitude of structural and functional brain changes, and prior classification models have had similar findings [Valfrè, W. et al. 2008; Schmitz, N. et al. 2008; Maleki, N. at al. 2012; Schwedt, T.J. et al. 2015; Gaw, N. et al. 2018; Magon, S. et al. 2019; Lai, K. L. et al. 2020; Mu, J. et al. 2020].

Study Limitations: We assumed heterogeneity amongst the healthy control cohort, and this assumption served as justification for developing a healthy core. However, a limitation of this study is that we are only partially able to describe the heterogeneity using the non-imaging data that were collected, such as the medical center from which a participant was enrolled, their sex, and age. Future studies should more deeply phenotype the healthy control subjects in search of sources of heterogeneity that might associate with

well but you can already do that with descriptive statistics of brain regions volumes, areas and thicknesses! much simpler than doing ML models!

differences in brain structure. Also, it should be noted that imaging data used in the analyses reported herein

this should already be done for this paper! use metrics such as mean, std, coefficient of variation, medians, IQR, even skewness and kurtosis

were also used in prior research on classification models for migraine [Schwedt, T.J. et al. 2015; Gaw, N.

and even boxplots or rather violin plots ... this is actually what you should do before running any ML model, statistics of your variables!

et al. 2018]. Future studies will continue to validate the migraine classification models reported herein and test the value of using a healthy core when developing new classification models.

In conclusion, the utilization of a healthy core can increase the accuracy and generalizability of brain imaging-based classification models for EM and CM. Inclusion of a healthy core addresses intrinsic heterogeneity that exists within a healthy control cohort and in multicenter studies, even when stringent participant eligibility criteria and methodology are used.

Funding Statement: This work was supported by the United States Department of Defense W81XWH-15-1-0286, National Institutes of Health K23NS070891, and internal funds from the Mayo Clinic.

References

need to review and complete the references

Ashina S, Bentivegna E, Martelletti P, & Eikermann-Haerter K, Structural and functional brain changes in migraine. Pain and Therapy. 2021; 10(1): 211-223.

Baochen S, Correlation Alignment for Domain Adaptation. PhD thesis, University of Massachusetts Lowell, 8 2016

any page numbers were the specific content may be found?

Chen AA, Beer JC, Tustison NJ, Cook PA, Shinohara RT, Shou H, & Alzheimer's Disease Neuroimaging Initiative, Mitigating site effects in covariance for machine learning in neuroimaging data. Human brain mapping. 2022; 43(4): 1179-1195.

Chong CD, Gaw N, Fu Y, Li J, Wu T, & Schwedt TJ, Migraine classification using magnetic resonance imaging resting-state functional connectivity data. Cephalalgia. 2017;37(9): 828-844.

Classification Committee of the International Headache Society. The International Classification of Headache Disorders, 3rd edition. Cephalalgia. 2018; 38: 1-211.

Classification Committee of the International Headache Society. The International Classification of Headache Disorders, 3rd edition (beta version). Cephalalgia. 2013;33:629-808.

Csurka G, Domain adaptation for visual applications: A comprehensive survey. arXiv preprint arXiv:1702.05374 [Preprint]. 2017.

Cui P, Hu W, & Zhu J, Calibrated reliable regression using maximum mean discrepancy. Advances in Neural Information Processing Systems. 2020; 33.

Dale AM, Fischl B, & Sereno MI, Cortical surface-based analysis: I. Segmentation and surface reconstruction. Neuroimage. 1999; 9(2): 179-194.

Duan L, Tsang IW, Xu D, & Maybank SJ, Domain transfer svm for video concept detection. In 2009 IEEE Conference on Computer Vision and Pattern Recognition, 2009; 1375-1381.

Eck J, Richter M, Straube T, Miltner WH, & Weiss T, Affective brain regions are activated during the processing of pain-related words in migraine patients. Pain. 2011; 152(5): 1104-1113.

Firnkorn D, Ganzinger M, Muley T, Thomas M, & Knaup P, A generic data harmonization process for cross-linked research and network interaction. Methods of information in medicine. 2015; 54(05): 455-460.

Fischl B, Liu A, & Dale AM, Automated manifold surgery: constructing geometrically accurate and topologically correct models of the human cerebral cortex. IEEE transactions on medical imaging. 2001; 20(1): 70-80.

Fischl B, Salat DH, Busa E, Albert M, Dieterich M, Haselgrove C et al., Whole brain segmentation: automated labeling of neuroanatomical structures in the human brain. Neuron. 2002; 33(3): 341-355.

Epstein EG, Whitehead PB, Prompahakul C, Thacker LR, & Hamric AB, Enhancing understanding of moral distress: the measure of moral distress for health care professionals. AJOB empirical bioethics. 2019; 10(2): 113-124.

Gaw N, Schwedt TJ, Chong CD, Wu T, & Li J, A clinical decision support system using multi-modality imaging data for disease diagnosis. IISE Transactions on Healthcare Systems Engineering. 2018; 8(1): 36-46.

Gong B, Shi Y, Sha F, & Grauman K, Geodesic flow kernel for unsupervised domain adaptation. In 2012 IEEE Conference on Computer Vision and Pattern Recognition. 2012, June: 2066-2073.

Gretton A, Borgwardt KM, Rasch MJ, Schölkopf B, & Smola A, A kernel two-sample test. The Journal of Machine Learning Research. 2012; 13(1): 723-773.

Hutson, M, Artificial intelligence faces reproducibility crisis. 2018. Is this a book? an article?

Kirchler M, Khorasani S, Kloft M, & Lippert C, Two-sample testing using deep learning. In International Conference on Artificial Intelligence and Statistics. 2020, June; 1387-1398. PMLR. what does it mean the acronym?

Lai KL, Niddam DM, Fuh JL, Chen WT, Wu JC, & Wang SJ, Cortical morphological changes in chronic migraine in a Taiwanese cohort: surface-and voxel-based analyses. Cephalalgia. 2020; 40(6): 575-585.

Lee MJ, Park BY, Cho S, Park H, Kim ST, & Chung CS, Dynamic functional connectivity of the migraine brain: a resting-state functional magnetic resonance imaging study. Pain, 2019; 160(12): 2776-2786.

Lo Buono V, Bonanno L, Corallo F, Pisani LR, Lo Presti R, Grugno R et al., Functional connectivity and cognitive impairment in migraine with and without aura. The journal of headache and pain. 2017; 18(1): 1-6.

Long M, Cao Y, Wang J, & Jordan M, Learning transferable features with deep adaptation networks. In International conference on machine learning. 2015, June; 97-105. PMLR.

Long M, Wang J, Ding G, Sun J, & Yu PS, Transfer feature learning with joint distribution adaptation. In Proceedings of the IEEE international conference on computer vision, 2013; 2200-2207.

Ma D, Popuri K, Bhalla M, Sangha O, Lu D, Cao J et al., & Alzheimer's Disease Neuroimaging Initiative, Quantitative assessment of field strength, total intracranial volume, sex, and age effects on the goodness of harmonization for volumetric analysis on the ADNI database. Human brain mapping. 2019; 40(5): 1507-1527.

Magon S, May A, Stankewitz A, Goadsby PJ, Schankin C, Ashina M et al., Cortical abnormalities in episodic migraine: a multi-center 3T MRI study. Cephalalgia. 2019; 39(5): 665-673.

Maleki N, Becerra L, Brawn J, Bigal M, Burstein R, & Borsook D, Concurrent functional and structural cortical alterations in migraine. Cephalalgia. 2012; 32(8): 607-620.

Matasci G, Volpi M, Kanevski M, Bruzzone L, & Tuia D, Semisupervised transfer component analysis for domain adaptation in remote sensing image classification. IEEE Transactions on Geoscience and Remote Sensing. 2015; 53(7): 3550-3564.

Meylakh N, Marciszewski KK, Di Pietro F, Macefield VG, Macey PM, & Henderson LA, Deep in the brain: changes in subcortical function immediately preceding a migraine attack. Human brain mapping. 2018; 39(6): 2651-2663.

Mu J, Chen T, Quan S, Wang C, Zhao L, & Liu J, Neuroimaging features of whole- brain functional connectivity predict attack frequency of migraine. Human brain mapping. 2020; 41(4): 984-993.

Scheepens DS, van Waarde JA, Lok A, de Vries G, Denys DA, & van Wingen GA, The link between structural and functional brain abnormalities in depression: a systematic review of Multimodal Neuroimaging Studies. Frontiers in psychiatry. 2020; 11: 485.

Schmitz N, Admiraal- Behloul F, Arkink EB, Kruit MC, Schoonman GG, Ferrari MD, & Van Buchem MA, Attack frequency and disease duration as indicators for brain damage in migraine. Headache: The Journal of Head and Face Pain. 2008; 48(7): 1044-1055.

Schwedt TJ, Chong CD, Wu T, Gaw N, Fu Y, & Li J, Accurate classification of chronic migraine via brain magnetic resonance imaging. Headache: The Journal of Head and Face Pain. 2015; 55(6): 762-777.

Schwedt TJ, Si B, Li J, Wu T, & Chong CD, Migraine subclassification via a data- driven automated approach using multimodality factor mixture modeling of brain structure measurements. Headache: The Journal of Head and Face Pain. 2017; 57(7): 1051-1064.

Ségonne F, Dale AM, Busa E, Glessner M, Salat D, Hahn HK, & Fischl B, A hybrid approach to the skull stripping problem in MRI. Neuroimage. 2004; 22(3): 1060-1075.

Sinno JP, James TT, Ivor W, & Qiang Y, Domain adaptation via transfer component analysis. Transactions on Neural Networks. 2011; 22(2): 199–210.

Stupple A, Singerman D & Celi LA, The reproducibility crisis in the age of digital medicine. NPJ digital medicine. 2019; 2(1): 1-3.

Tang H, Chen K, & Jia K, Unsupervised domain adaptation via structurally regularized deep clustering. In Proceedings of the IEEE/CVF conference on computer vision and pattern recognition. 2020; 8725-8735.

Tzeng E, Hoffman J, Saenko K, & Darrell T, Adversarial discriminative domain adaptation. In Proceedings of the IEEE conference on computer vision and pattern recognition. 2017; 7167-7176.

Valfrè W, Rainero I, Bergui M, & Pinessi L, Voxel- based morphometry reveals gray matter abnormalities in migraine. Headache: The Journal of Head and Face Pain. 2008; 48(1): 109-117.

Van der Maaten L, & Hinton G, Visualizing data using t-SNE. Journal of machine learning research. 2008; 9(11).

Wei HL, Chen J, Chen YC, Yu YS, Guo X, Zhou GP et al., Impaired effective functional connectivity of the sensorimotor network in interictal episodic migraineurs without aura. The Journal of Headache and Pain. 2020; 21(1): 1-9.

Yan H, Ding Y, Li P, Wang Q, Xu Y, & Zuo W, Mind the class weight bias: Weighted maximum mean discrepancy for unsupervised domain adaptation. In Proceedings of the IEEE Conference on Computer Vision and Pattern Recognition. 2017; 2272-2281.

Yang H, Zhang J, Liu Q, & Wang Y, Multimodal MRI-based classification of migraine: using deep learning convolutional neural network. Biomedical engineering online. 2018; 17(1): 1-14.

Yao Y, & Doretto G, Boosting for transfer learning with multiple sources. In 2010 IEEE computer society conference on computer vision and pattern recognition. 2010, June; 1855-1862.

Yu M, Linn KA, Cook PA, Phillips ML, McInnis M, Fava M, et al., Statistical harmonization corrects site effects in functional connectivity measurements from multi- site fMRI data. Human brain mapping. 2018; 39(11): 4213-4227.

Zhang Q, Wu Q, Zhang J, He L, Huang J, Zhang J et al., Discriminative analysis of migraine without aura: using functional and structural MRI with a multi-feature classification approach. PloS one. 2016; 11(9): e0163875.

Zhang W, & Wu D, Discriminative joint probability maximum mean discrepancy (DJP-MMD) for domain adaptation. In 2020 International Joint Conference on Neural Networks (IJCNN). 2020, July; 1-8.

Appendix

The appendix should be mentioned in the main text.

The proposed method, a unified framework of GFK+MMD

The Maximum Mean Discrepancy (MMD) is a single statistic to measure the closeness between two distributions $\mathbb{P}$ and $\mathbb{Q}$ from two datasets.

what does sup mean?

$$MMD(\mathbb{P},\mathbb{Q};\mathcal{F}) \coloneqq \sup_{f\in\mathcal{F}} |\,\mathbb{E}[f(DS_1)] - \mathbb{E}[f(DS_2)]\,| \quad (1)$$

where $\mathcal{F}$ is a set containing all continuous functions [Gretton, A et al. 2012]

The Geodesic Flow Kernel [Gong, B et al. 2012], also known as GFK, has utilized Grassmann manifolds and enabled the construction of physically meaningful new representations between two datasets. GFK explores several feature representations between two datasets and generates a number of transformations that characterize changes in geometric and statistical properties from one dataset to another dataset. Since this approach explores several statistically meaningful feature representations between two datasets, the advantage of several representations by GFK can be used for capturing data variabilities instead of relying on a single snapshot of each dataset. MMD is utilized as the tool to compare those feature representations.

Algorithm 1: a proposed algorithm for Healthy Core

Input: Data $\mathbf{X}_1$ and Data $\mathbf{X}_2$;
Output: A single coreset $\{\mathbf{X}_{1c}, \mathbf{X}_{2c}\}$

explain how GFK is used here.

begin
  repeat
    Iteration i =1 to $\mathbf{n}_1$ samples from $\mathbf{X}_{1c}$

what does G mean?

      Given $\mathbf{X}_{2c}$ Construct MMD matrix $\left\{G(\mathbf{X}_{1C_i}), G(\mathbf{X}_{2C})\right\}$
    Iteration j =1 to $\mathbf{n}_2$ samples from $\mathbf{X}_{2c}$
      Given $\mathbf{X}_{1c}$ Construct MMD matrix $\left\{G(\mathbf{X}_{1C}), G(\mathbf{X}_{2C_j})\right\}$
    Compute rank by MMD and select sample to maximize decreasing the average MMD along t
  until *Convergence (optimal point)*
  Return

Click here to access/download
Supporting Information
Data Availability Statements.pdf

Click here to access/download
Supporting Information
STROBE-checklist-v4-combined-PlosMedicine.docx

Click here to access/download
Supporting Information
IRB Approval 2010.pdf

Click here to access/download

Supporting Information

dod fp irb approval.pdf

Click here to access/download
Supporting Information
functional networks in migraine IRB approval (1).pdf

Click here to access/download
Supporting Information
IRB approval migraine vs PTH.pdf

Click here to access/download
Supporting Information
IRB approval PPTH vs migraine.pdf
