# Supplementary material for: Healthy core: Harmonizing brain MRI for supporting multicenter migraine classification studies
Source: PLoS One. 2024 Dec 31;19(12):e0288300. doi: 10.1371/journal.pone.0288300 (PMC11687649; doi:10.1371/journal.pone.0288300)
Supplement: Attachment — Submitted filename: PONE-D-23-16414_rev1_20230716.docx [file pone.0288300.s003.docx]

This research study suggests a strategy designed to enhance the accuracy and generalizability of machine learning models applied in multicenter imaging studies. The ultimate objective is to classify individual migraine patients and healthy controls using brain MRI data. Authors employed the Maximum Mean Discrepancy (MMD) in Geodesic Flow Kernel (GFK) space, to harmonize data from two sources. Their approach consisted of identifying a group of homogeneously healthy controls, called the "healthy core". The authors suggest this methodology effectively mitigated typical confounding factors inherent in multicenter studies such as participant characteristics variations, MRI scanner disparities, and divergent imaging acquisition protocols. The study utilized two datasets: one with 120 individuals (comprising 66 migraine patients and 54 controls) and another with 76 (consisting of 34 migraine patients and 42 controls). The implementation of the "healthy core" concept reportedly led to a 25% enhancement in the accuracy of the classification models for both episodic and chronic migraine patients. The authors have proficiently conveyed their findings and offered comprehensive information about their experiments.

The primary aim of my comments (both major and minor) is to help the authors advance their study to the next level. This is achievable by addressing currently ambiguous aspects, thus enhancing the study's scientific merit and improving its readability and flow.

Major concerns:

1. The authors applied the Maximum Mean Discrepancy (MMD) and Geodesic Flow Kernel (GFK) techniques to construct the "healthy core". The fundamental concept of MMD is the mapping of data samples into a high-dimensional feature space, typically via a kernel function, followed by a comparison of mean features in that space. Identical distributions yield identical mean features. The study used GFK space to represent the data, a technique prevalent in domain adaptation, which involves knowledge transfer from a source domain (one center's MRI data) to a target domain (another center's MRI data). A continuous flow of subspaces linking the source and target domains was created using GFK, thereby bridging the gap between datasets from different centers.
   a. In this study, MMD was utilized to compare two datasets in the GFK space, allowing for a comparison of the underlying probability distributions of these datasets. The choice of kernel in MMD (linear, polynomial, radial, gaussian, Laplacian, etc.) and the setting of its parameters can dramatically influence results. Furthermore, both MMD and GFK's performance are heavily contingent upon the kernel and its parameters. Choosing an appropriate kernel can be difficult and very challenging. The authors would do well to elaborate on this aspect, shedding light on the kernel selection process and the parameters used in their study.
   b. While MMD allows for an overall comparison of two datasets' distributions, it might lack sensitivity towards specific features of interest in the MRI images. Likewise, GFK primarily focuses on the broader shifts between domains, possibly neglecting nuanced differences. Were there any tests conducted to assess the specificity of these methods?
2. Sample size plays a crucial role in the effectiveness and reliability of statistical techniques such as the MMD. If the sample sizes are too small, the estimation of the underlying distribution of data could be imprecise. This can impact the results of the MMD in two ways: statistical power and overfitting. Given the sample sizes (120 and 75), while not large, they are not extremely small either. In such circumstances t's often beneficial to perform some form of validation or sensitivity analysis to assess the robustness of results to variations in data. For example, resampling techniques like bootstrapping or cross-validation could be used to assess the stability of the MMD measure with respect to the sample size. Could authors comment on that?
3. The differences between the HC datasets are quite confusing and pose several questions. The authors reported on pages 9-10 that "…..the mean of area, thickness, and volume features significantly differ between the HCs in the two datasets with a p-value<0.01 before the False Discovery Rate (FDR) correction.". Dataset 1 comprised 54 HCs (mean age, including patients, 36±11), and dataset 2 comprised 42 HCs (mean age, including patients, 38±10). MRI data were acquired on different models of Siemens scanners, and the acquisition parameters were almost identical. The data analysis was carried out using FreesurferV5.3. Figure 2 illustrates the mean differences in volume, thickness, and surface area between HCs in the datasets for 68 parcellation in the brain, based on FreeSurferV5.3 output.
   a. On a practical note, there seems to be no strong reason for such differences between these two HC datasets. Even if the scanner vendors are different (e.g., Siemens vs. GE or Siemens vs. Philips, etc.), we might expect some difference, but it should be minimal. How did the authors manage to capture these differences? Were there any quality control processes in place? Were there any manual edits after the FreeSurfer automatic pipeline? I'm curious about how the authors can explain these significant differences in morphometric measurements between nearly identical datasets and their scanners. The observed differences are, for the most part, not scanner related. Are they artificially created to conduct the experiment?
   b. FreeSurfer computes cortical gray matter volume by multiplying cortical thickness and surface area (gray matter volume = cortical thickness * surface area). Figure 2 shows that the difference is positive for volume and area (i.e., larger in one of the data sets compared to the other), but it is both positive and negative for thickness.

In other words, if volume is larger in DS1 compared to DS2 that means either area or thickness or both are larger in DS1 compared to DS2. This means that the differences between the features in both datasets do not align with the expected mathematical relationship between thickness, volume, and area. Could the authors provide their thoughts on this? It is likely that these differences are due to low-quality MRI scans, but more importantly, they might be attributed to inaccurate segmentation and cortical parcellation using an outdated version of FreeSurfer.

Minor comments:

1. Title: The “Healthy Core” is an important innovative step in this study. The authors might consider tweaking their study title to incorporate this terminology.
2. The abstract is generally well-written but could benefit from minor improvements:
   a. On Page #2 first two lines, authors mentioned: “Multicenter and multi-scanner imaging studies might be needed to provide sample sizes large enough for
      developing accurate predictive models”. The sentence is scientifically accurate, clear, and concise. However, there's a minor grammatical error that can be corrected for better readability. Instead of using the phrase "might be needed to provide sample sizes large enough for", please consider rephrasing it as "may be necessary to ensure sufficiently large sample sizes for". The revised sentence could be: "Multicenter and multi-scanner imaging studies may be necessary to ensure sufficiently large sample sizes for developing accurate predictive models." The change from "might be" to "may be" is minor and subjective, but it can make the sentence sound slightly more formal or academic. The phrase "ensure sufficiently large sample sizes" also provides a bit more clarity than "provide sample sizes large enough".
   b. The last sentence is a fragment ("Extensive experimental results show that the utilization of a healthy core.") and should be revised for completeness. Also, the text could be better organized for clarity. For example, the information about the datasets could be introduced earlier to provide context for the discussion on the "healthy core" step.
   c. The abstract provides a good summary of the research but could be clearer on some aspects. The abstract should explain why a homogeneous control group is better and what exactly "homogeneous" means in this context. It should also detail how the MMD is used in GFK space to identify a "healthy core". Additionally, the abstract could strengthen its scientific merit by providing more specific information on the "extensive experimental results," rather than a general statement.
3. The introduction is generally well-written with minor issues:
   a. There are some long sentences that might be split for better readability. Examples are too many:
      i. “Machine learning models trained to automatically classify individuals with migraine from healthy controls (HCs) using functional magnetic resonance imaging (fMRI) and structural MRI data have shown good utility for discriminating those with migraine from HCs.”
      ii. “Yet, the generalization of the algorithms might be poor; that is, the classification model developed using one dataset may not perform as accurately on a separate dataset.”
      iii. "Harmonization in clinical research has been focusing on defining, reviewing, and standardizing common data elements [Firnkorn, D et al. 2015, Yu, M. et al. (2018), Ma, D. et al. (2019), Chen, A. A. et al. (2022)]."
      iv. "This is known as data curation - a strategy to combine the data from different sources to improve the generalizability of classification models."
      v. "Yet, from a data-driven aspect, the data from different sources may inherently exhibit different characteristics due to several reasons, such as differences in patient populations from which research participants are identified and the methods by which data are acquired."
      vi. "In machine learning research, extensive efforts are dedicated to transforming the data from different sources into a common feature space for evaluating similarities from multiple sources or adapting them thoroughly."

These are a few examples of sentences that are long and contain several ideas or points. They could be broken down into shorter sentences to make them easier to read and understand.

b. There is inconsistent use of periods in the reference citations which could lead to confusion.
c. The introduction provides a good background of the problem and proposes a novel concept “the healthy core”, but it would benefit from more detailed explanation of why existing Domain Adaptation strategies are insufficient and how the "healthy core" concept improves upon them. Additionally, some technical terms (like "Domain Adaptation") are introduced without a clear definition, which might confuse readers not familiar with the terminology. Lastly, it would be beneficial to elaborate on the procedure of creating a "healthy core", even if it is explained later, to give the reader a clearer idea of what it entails.

4. Methods: The methodology for data collection is clearly outlined, and the comprehensive nature of the data collected lends credibility to the research. However:
   a. some more contexts could be provided to explain the relevance or importance of each specific type of data collected (e.g., BDI-II scores, STAI scores, etc.).
   b. The list of exclusion criteria could be better structured to enhance readability.
   c. The participant selection criteria are well-detailed, providing a clear picture of the study population. One point might be useful to specify why the age range was specifically 18 to 65 years.
   d. The paragraph “data collection” is well-structured and clear, but it might benefit from the use of sub-headings or bullet points for the details of each scanner's specifications to enhance readability. Authors may consider including this information in a table or in supplementary material.
   e. The relationship between DS1 and DS2 is unclear; it is uncertain whether they are two independent datasets or if DS2 is a subset of DS1, especially since both datasets contain patients scanned at the same center.
   f. The sentence on page 6, "T1 images were used for ruling out gross structural abnormalities," could be rephrased as follows: "T1 images were employed to exclude any significant or clearly visible structural abnormalities”.
   g. The sentence on page 6, “Data included in this analysis have been utilized in prior analyses [Schwedt, T.J., 2015 and Chong, C. D. 2017]”. Both datasets were utilized or one or subset of participants? It seems MRI T1-weighted images of 55 migraine patients and 58 healthy controls were used in that study, this is not the same number of participants in this current study. Please clarify.
   h. The sentence on page 7, “In order to avoid the confounding of multiple cohort differences, the mean and
      variance differences were removed from each cohort to identify the remaining differences”:
      i. Does the author intend to use “in order to avoid” or “in order to mitigate” in this context?
      ii. The sentence could benefit from additional context and clarity. In statistics and data analysis, confounding often refers to the distortion of an estimated effect caused by an extraneous variable. In this case, it seems that the sentence is referring to the removal of mean and variance differences between cohorts to identify other (residual) differences more accurately. Please clarify.
   i. In the paragraph on page 8, the authors state, “We hypothesize that using a healthy core, a homogeneous subset of HCs from different sources, can help improve the generalization of the machine learning model for classifying migraine. This strategy addresses the heterogeneity of the HCs within and across the datasets and bridges the differences between the datasets.” This hypothesis should be introduced earlier in the manuscript, preferably in the abstract. This will give the reader a better opportunity to understand the manuscript and prepare for what is next.
      i. The term 'hypothesis' is mentioned multiple times throughout the text; if there are multiple hypotheses, these should be presented earlier to familiarize the reader with the authors' intentions. It seems the authors might be confusing a hypothesis with a belief or expectation. A hypothesis is a testable proposition or prediction that is based on known information and that forms the basis for further investigation, while a belief is a personal view, opinion or conviction that does not necessarily have to be testable.

For instance, on page 7, the statement: “To capture the inherent variabilities, we hypothesize that a Geodesic Flow Kernel (GFK), which utilizes geometric manifolds termed Grassmann, may help," reads more like an assertion rather than a formal hypothesis. It could be rephrased as follows: "To capture the inherent variabilities, we utilized a Geodesic Flow Kernel (GFK) that employs geometric manifolds, termed Grassmann." Similarly, on page 4, the authors state: "We hypothesized that, compared to classification models developed without the use of a healthy core, imaging-based classification models for migraine developed with the use of a healthy core would have higher classification accuracy when applied to an unseen dataset." This also reads more like a belief rather than an established hypothesis. Please clarify.

j. On page 8, the authors mention, "To achieve reliable results for the relatively small sample sizes, experiments are repeated under five different random scenarios (cross-validated) to build classifiers and report average performances." However, regardless of the number of times experiments are conducted, the small sample size presents a significant challenge in this study. This is a crucial limitation that needs to be clearly addressed in the limitations section, along with all the other concerns I have raised in my review. I would encourage the authors to further elaborate on these limitations in that section.

5. The results: Please address the following points:
   a. On page 9, I would recommend incorporating gender and age information (mean and standard deviation) into Tables 1 and 2 for improved clarity.
   b. On page 10, I would suggest removing Table 3 and integrating the information directly into the text. This is because the table, as it currently stands, does not seem to add significant value to the content.
   c. On page 10, the authors mention: “This result confirms that there are mean differences between the two cohorts for each modality.” Could it be that the authors meant “features” instead of “modality”? Please clarify.
   d. Once again, on page 10, the authors state, “This result confirms that there are mean differences between the two cohorts for each modality” I have previously commented on this difference in the methods section and find it essential to emphasize my concern here, as the difference is perplexing. The authors interpret this difference as indicating that the healthy control groups are heterogeneous, which they suggest necessitates the establishment of a “healthy core.” However, there is no explanation as to why this large difference emerges between two groups of healthy controls with nearly identical age ranges (37 and 38 years old), scanned under nearly identical scanner settings, and whose data was processed using the same version of FreeSurferV5.3. An explanation for this difference is crucial. Is it artificially created and designed to facilitate the experiment? Could it be due to poor quality MRI images that required manual edits from different users? While differences between centers related to MRI processing are expected, the scale of the discrepancy observed here is unusually significant. Are these differences consistent with similar differences introduced in other multi center studies?
   e. In Table 5, the header “DS1 vs DS2” is mentioned three times, which leads to confusion. Could you please clarify?
   f. On pages 17-18, the figures presented are simple scatter plots, the clarity of which could be improved. Also, please consider adding a legend to specify the meaning of the red and blue dots.
6. Discussion and Conclusion:
   a. Please consider dividing the “discussion and conclusion” into two separate sections.
   b. In the first paragraph of page 19, it's critical for the authors to define what they mean by “heterogeneous” with regard to the healthy control groups. This clarification is particularly important in providing a rationale for constructing the “healthy core.” The authors should elaborate on why their healthy control groups display significant differences in the mean values of features such as thickness, volume, and area. Without this explanation, it could be inferred that these differences were artificially manufactured to facilitate the experiment. While we expect differences in mean features among multi-center data, the large discrepancy observed in the authors' settings is quite puzzling.
7. Limitations: While reviewing the manuscript, I found many limitations in this study. I encourage authors to think about the following points:
   a. Please consider creating a section with the header “Limitations”.
   b. Consider the effect of sample sizes on the MMD calculation.
   c. MMD relies on the assumption that the distributions being compared are homogeneous. If the data have underlying heterogeneities, MMD might not accurately represent the closeness of these distributions.
   d. The use of MMD and GFK can be computationally intensive, especially when dealing with large datasets. This could potentially limit the scalability of the proposed framework, particularly in real-world settings where datasets can be very large.
   e. The GFK method is sensitive to the choice of parameters, such as the number of subspaces in the Grassmann manifold. Incorrect parameter selection can significantly impact the performance of the method.
   f. Another limitation to consider is that GFK creates a new feature space that captures the gradual change from one dataset to another. While this feature space can be statistically meaningful, it might not be directly interpretable in the context of the original data. This could pose challenges in understanding and interpreting the results.
   g. Both MMD and GFK are dependent on the quality of the data. Issues like missing data, noise, and errors can impact their performance. Furthermore, these issues can affect the quality of the derived “healthy core.”.

I hope the points raised in this review will help guide the authors in refining their study further. I believe that addressing these concerns will elevate the quality of their research and help bring their work to the next level. I look forward to seeing the revised manuscript.
