# Supplementary material for: Healthy core: Harmonizing brain MRI for supporting multicenter migraine classification studies
Source: PLoS One. 2024 Dec 31;19(12):e0288300. doi: 10.1371/journal.pone.0288300 (PMC11687649; doi:10.1371/journal.pone.0288300)
Supplement: Attachment — Submitted filename: Response letter to PlosOne_231109_Final.docx [file pone.0288300.s005.docx]

November 10, 2023

Dr. Lorenzo Faggioni

Academic Editor

PLOS ONE

**Reference**

Harmonizing Healthy Cohorts to Support Multicenter Studies on Migraine Classification using Brain MRI Data

Dear Dr. Lancaster,

Thank you for offering us the opportunity to respond to the comments on our revised paper titled “Harmonizing Healthy Cohorts to Support Multicenter Studies on Migraine Classification using Brain MRI Data” (Reference: PONE-D-23-16414). In this letter, we have outlined our responses to the comments from Reviewers #1 and #2.

We found the reviews of our manuscript insightful and helpful in improving the paper. We would like to extend our thanks to the reviewers and the editorial team.

Sincerely,

Hyunsoo Yoon, Ph.D.

Assistant Professor, Department of Industrial Engineering

College of Engineering, Yonsei University

Tel: +82-2-2123-4018

E-mail: [hs.yoon@yonsei.ac.kr](mailto:hs.yoon@yonsei.ac.kr)

Enclosures

**Comments from Reviewer #1:**

**Concern #1.**

The authors applied the Maximum Mean Discrepancy (MMD) and Geodesic Flow Kernel (GFK) techniques to construct the "healthy core". The fundamental concept of MMD is the mapping of data samples into a high-dimensional feature space, typically via a kernel function, followed by a comparison of mean features in that space. Identical distributions yield identical mean features. The study used GFK space to represent the data, a technique prevalent in domain adaptation, which involves knowledge transfer from a source domain (one center's MRI data) to a target domain (another center's MRI data). A continuous flow of subspaces linking the source and target domains was created using GFK, thereby bridging the gap between datasets from different centers.

**Concern # 1 - Comment #1-a.**

In this study, MMD was utilized to compare two datasets in the GFK space, allowing for a comparison of the underlying probability distributions of these datasets. The choice of kernel in MMD (linear, polynomial, radial, gaussian, Laplacian, etc.) and the setting of its parameters can dramatically influence results. Furthermore, both MMD and GFK's performance are heavily contingent upon the kernel and its parameters. Choosing an appropriate kernel can be difficult and very challenging. The authors would do well to elaborate on this aspect, shedding light on the kernel selection process and the parameters used in their study.

**Response:** Thank you for the comment, which we have also addressed in Concern #10.

We appreciate your feedback and acknowledge the significance of the choice of kernel and its parameters in the MMD technique. In our study, we opted to utilize the fundamental, parameter-free method as introduced in MMD [Gretton, Arthur, et al. 2012]. This choice was made by two key factors. Firstly, considering the size of our sample in the medical applications, we sought to minimize the introduction of additional parameters, which could lead to overfitting with limited data. Secondly, as this marks our initial exploration into data harmonization, we aimed to establish a strong foundation before incorporating more complex variants.

In our forthcoming work, we plan to implement and evaluate the performance of MMD variants that incorporate parameters. This will allow us to assess the impact of various kernels and parameter settings on our data harmonization process. We have updated our conclusion to reflect this plan, ensuring that we address the concerns related to kernel selection and parameter settings in more detail.

[Reference: Gretton, Arthur, et al. "A kernel two-sample test." The Journal of Machine Learning Research 13.1 (2012): 723-773.]

**Concern # 1 - Comment #1-b.**

While MMD allows for an overall comparison of two datasets' distributions, it might lack sensitivity towards specific features of interest in the MRI images. Likewise, GFK primarily focuses on the broader shifts between domains, possibly neglecting nuanced differences. Were there any tests conducted to assess the specificity of these methods?

**Response:**

Thank you for your valuable input. The GFK approach was adopted for its computational simplicity and the reduction in the need for extensive parameter tuning, as opposed to Gopalan et al.'s (2011) method. The original GFK technique is straightforward to implement, as it does not require cross-validation for subspace quantity. Leveraging the kernel trick, GFK can capture an infinite number of subspaces. In our proposed modification, we avoid the need for parameter selection concerning subspaces and kernels.

Regarding the specificity of MMD and GFK, we acknowledge the traditional use of MMD for assessing dataset distribution and GFK's focus on understanding and describing the characteristics of datasets. However, our study's primary goal is not to adapt dataset A to dataset B or to identify differences between A and B, whether significant or nuanced.

Our main objective is to identify the core members within the combined dataset (A+B) that exhibit greater discriminatory power. Therefore, differentiating A from B is not the central focus of this study. Instead, we have assessed the performance of the healthy core. Our hypothesis is that, even though all samples are labeled as healthy, the healthy core differs from the healthy non-core.

We conducted a univariate analysis to investigate the mean differences between two healthy control groups and within the healthy core. The univariate analysis aimed to examine the mean differences between two healthy control groups. Additionally, we conducted a univariate analysis to explore the mean differences within the healthy core, randomly splitting it into two groups. This process was repeated 100 times, and the resulting average values were reported.

Univariate analysis for mean difference between two healthy controls.

| Modality | p-values | $\mu_{D2HC}-\mu_{D1HC}$ | False Discovery Rate |
| --- | --- | --- | --- |
| Area features | 0.0087 | 119.0078 | 0.0204 |
| Thickness features | 0.0136 | -0.0275 | 0.0204 |
| Volume features | 0.0302 | 277.4083 | 0.0302 |

Univariate analysis for mean difference within health core. (randomly splitting it into two core groups). Repeating this 100 times. Reported averaged values are reported.

| Modality | p-values | $\mu_{HC (random)}-\mu_{HC (random)}$ | False Discovery Rate |
| --- | --- | --- | --- |
| Area features | 0.0591 | 108.7624 | 0.0701 |
| Thickness features | 0.0702 | -0.0231 | 0.0783 |
| Volume features | 0.0881 | 237.8323 | 0.0881 |

Domain adaptation for object recognition: An unsupervised approach. In 2011 international conference on computer vision (pp. 999-1006). IEEE.

**Concern & comment #2.**

Sample size plays a crucial role in the effectiveness and reliability of statistical techniques such as the MMD. If the sample sizes are too small, the estimation of the underlying distribution of data could be imprecise. This can impact the results of the MMD in two ways: statistical power and overfitting. Given the sample sizes (120 and 75), while not large, they are not extremely small either. In such circumstances t's often beneficial to perform some form of validation or sensitivity analysis to assess the robustness of results to variations in data. For example, resampling techniques like bootstrapping or cross-validation could be used to assess the stability of the MMD measure with respect to the sample size. Could authors comment on that?

**Response:** Thank you for the comment. Addressed in Concern # 10.

**Concern #3.**

The differences between the HC datasets are quite confusing and pose several questions. The authors reported on pages 9-10 that "…..the mean of area, thickness, and volume features significantly differ between the HCs in the two datasets with a p-value<0.01 before the False Discovery Rate (FDR) correction.". Dataset 1 comprised 54 HCs (mean age, including patients, 36±11), and dataset 2 comprised 42 HCs (mean age, including patients, 38±10). MRI data were acquired on different models of Siemens scanners, and the acquisition parameters were almost identical. The data analysis was carried out using FreesurferV5.3. Figure 2 illustrates the mean differences in volume, thickness, and surface area between HCs in the datasets for 68 parcellation in the brain, based on FreeSurferV5.3 output.

**Concern # 3 - Comment #3-a.**

On a practical note, there seems to be no strong reason for such differences between these two HC datasets. Even if the scanner vendors are different (e.g., Siemens vs. GE or Siemens vs. Philips, etc.), we might expect some difference, but it should be minimal. How did the authors manage to capture these differences? Were there any quality control processes in place? Were there any manual edits after the FreeSurfer automatic pipeline? I'm curious about how the authors can explain these significant differences in morphometric measurements between nearly identical datasets and their scanners. The observed differences are, for the most part, not scanner related. Are they artificially created to conduct the experiment? Patient heterogeneity and featurespace differences

**Response:** Thank you for your comment. We share your surprise regarding the observed differences in the healthy control (HC) datasets. Initially, we anticipated more pronounced imaging disparities between the HC and migraine cohorts scanned at different institutions, namely the Mayo Clinic and Washington University School of Medicine. Instead, we encountered significant dissimilarity between the DS1 and DS2 HC cohorts, both scanned at the Mayo Clinic but at different time points.

While we can only speculate at this point, we believe that upgrades in scanner operation and potential changes in coil performance during the period when these cohorts were scanned at the Mayo Clinic may have contributed to these findings.

In addition, our study's core motivation was to thoroughly analyze the dataset by comparing various combinations, such as A1+B1 with A2, A1 with A2, and A1+A2 with B1. Interestingly, the most substantial differences were observed when comparing A1+B1 with A2, where data were collected at different time points. To clarify, 'A' and 'B' represent datasets from different institutions, while '1' and '2' denote distinct time points within the same institution.

We recognize that several confounding factors may be at play, including temporal variations related to the software pipeline used in data processing and potential updates to the scanner software. Demographic differences could also contribute to the observed variations.

Our primary focus is to conduct a thorough investigation and gain a comprehensive understanding of these confounding factors. We acknowledge the need for further research to confidently identify the core factors responsible for the observed differences. We value your feedback and are dedicated to addressing these concerns in our research.

**Concern # 3 - Comment #3-b.**

FreeSurfer computes cortical gray matter volume by multiplying cortical thickness and surface area (gray matter volume = cortical thickness * surface area). Figure 2 shows that the difference is positive for volume and area (i.e., larger in one of the data sets compared to the other), but it is both positive and negative for thickness. In other words, if volume is larger in DS1 compared to DS2 that means either area or thickness or both are larger in DS1 compared to DS2. This means that the differences between the features in both datasets do not align with the expected mathematical relationship between thickness, volume, and area. Could the authors provide their thoughts on this? It is likely that these differences are due to low-quality MRI scans, but more importantly, they might be attributed to inaccurate segmentation and cortical parcellation using an outdated version of FreeSurfer.

**Response:** Thank you for your valuable feedback. We appreciate your observation and would like to clarify that the mean difference **scales** presented in Figure 2 are derived from two separate groups for each feature. These scales do not directly yield the gray matter volume using the formula gray matter volume = cortical thickness * surface area, as these are **differences between groups**. We firmly believe that our current calculations are accurate, validated through several verification steps. Additionally, we ensured consistency by verifying the results with the recent version of FreeSurfer. Hence, we do not believe that the significant differences observed can be attributed to inaccurate segmentation and cortical parcellation resulting from the use of an outdated version of FreeSurfer. We have confidence in the integrity of our findings, and we appreciate your diligence in reviewing our work.

**Concern & Comment # 4**

Title: The “Healthy Core” is an important innovative step in this study. The authors might consider tweaking their study title to incorporate this terminology.

**Response:** Thank you for the comment. The current title is “Harmonizing Healthy Cohorts to Support Multicenter Studies on Migraine Classification using Brain MRI Data”. Upon your suggestion, we revise our title as

“HealthyCore: Harmonizing Brain MRI for Supporting Multicenter Migraine Classification Studies”

**Concern 5**

The abstract is generally well-written but could benefit from minor improvements:

**Concern # 5 - Comment #5-a.**

On Page #2 first two lines, authors mentioned: “Multicenter and multi-scanner imaging studies might be needed to provide sample sizes large enough for
developing accurate predictive models”. The sentence is scientifically accurate, clear, and concise. However, there's a minor grammatical error that can be corrected for better readability. Instead of using the phrase "might be needed to provide sample sizes large enough for", please consider rephrasing it as "may be necessary to ensure sufficiently large sample sizes for". The revised sentence could be: "Multicenter and multi-scanner imaging studies may be necessary to ensure sufficiently large sample sizes for developing accurate predictive models." The change from "might be" to "may be" is minor and subjective, but it can make the sentence sound slightly more formal or academic. The phrase "ensure sufficiently large sample sizes" also provides a bit more clarity than "provide sample sizes large enough".

**Response:** Thank you for the comment. We have revised the aforementioned sentence as follows:

Multicenter and multi-scanner imaging studies may be necessary to ensure sufficiently large sample sizes for developing accurate predictive models.

**Concern # 5 - Comment #5-b.**

The last sentence is a fragment ("Extensive experimental results show that the utilization of a healthy core.") and should be revised for completeness. Also, the text could be better organized for clarity. For example, the information about the datasets could be introduced earlier to provide context for the discussion on the "healthy core" step.

**Response:** Thank you for the comment. We have corrected the aforementioned sentence as follows:

Homogeneous healthy controls play a vital role in mitigating unwanted heterogeneity, enabling the development of highly accurate classification models with improved performance on new datasets. Extensive experimental results underscore the benefits of leveraging a 'healthy core'. We utilized two datasets: one comprising 120 individuals (66 with migraine and 54 healthy controls), and another comprising 76 individuals (34 with migraine and 42 healthy controls). Notably, a homogeneous dataset derived from a cohort of healthy controls yielded a significant 25% accuracy improvement for both episodic and chronic migraineurs.

**Concern # 5 - Comment #5-c.**

The abstract provides a good summary of the research but could be clearer on some aspects. The abstract should explain why a homogeneous control group is better and what exactly "homogeneous" means in this context. It should also detail how the MMD is used in GFK space to identify a "healthy core". Additionally, the abstract could strengthen its scientific merit by providing more specific information on the "extensive experimental results," rather than a general statement.

**Response:** Thank you for the comment. We have revised the aforementioned sentences as follows:

This study focuses on enhancing generalizability in classifying individual migraine patients and healthy controls using brain MRI data through a data harmonization strategy. We propose identifying a 'healthy core'—a group of homogeneous healthy controls with similar characteristics—from multicenter studies. The Maximum Mean Discrepancy (MMD) in Geodesic Flow Kernel (GFK) space is employed to compare two datasets, capturing data variabilities and facilitating the identification of this 'healthy core’.

**Concern #6**

The introduction is generally well-written with minor issues. These are a few examples of sentences that are long and contain several ideas or points. They could be broken down into shorter sentences to make them easier to read and understand.

**Concern # 6 - Comment #6-a.**

There are some long sentences that might be split for better readability. Examples are too many:

i. “Machine learning models trained to automatically classify individuals with migraine from healthy controls (HCs) using functional magnetic resonance imaging (fMRI) and structural MRI data have shown good utility for discriminating those with migraine from HCs.”
ii. “Yet, the generalization of the algorithms might be poor; that is, the classification model developed using one dataset may not perform as accurately on a separate dataset.”
iii. "Harmonization in clinical research has been focusing on defining, reviewing, and standardizing common data elements [Firnkorn, D et al. 2015, Yu, M. et al. (2018), Ma, D. et al. (2019), Chen, A. A. et al. (2022)]."
iv. "This is known as data curation - a strategy to combine the data from different sources to improve the generalizability of classification models."
v. "Yet, from a data-driven aspect, the data from different sources may inherently exhibit different characteristics due to several reasons, such as differences in patient populations from which research participants are identified and the methods by which data are acquired."
vi. "In machine learning research, extensive efforts are dedicated to transforming the data from different sources into a common feature space for evaluating similarities from multiple sources or adapting them thoroughly."

**Response:** Thank you for the comment. We have revised the aforementioned sentences as follows:

Machine learning models, trained to automatically classify individuals with migraine from healthy controls (HCs) using functional magnetic resonance imaging (fMRI) and structural MRI data, have shown good utility for discriminating those with migraine from HCs.

However, the generalization of these algorithms might be poor. In other words, the classification model developed using one dataset may not perform as accurately on a separate dataset.

Harmonization in clinical research has been a focal point. It involves defining, reviewing, and standardizing common data elements.

This process is known as data curation. It involves a strategy to combine data from different sources, ultimately improving the generalizability of classification models.

Yet, from a data-driven aspect, the data from different sources may inherently exhibit different characteristics. This is due to several reasons, such as differences in patient populations from which research participants are identified and variations in the methods by which data are acquired.

In machine learning research, extensive efforts are dedicated to two main tasks. The first is transforming the data from different sources into a common feature space for evaluating similarities. The second involves adapting the data thoroughly to facilitate analysis.

**Concern # 6 - Comment #6-b.**

There is inconsistent use of periods in the reference citations which could lead to confusion.

**Response:** Thank you for the comment. We have revised the aforementioned sentences as follows:

[Eck, J. et al. (2011); Lo Buono et al. (2017); Meylakh, N. et al. (2018); Mu, J. et al. (2020); Wei H. et al. (2020); Scheepens, D. S. (2020); Ashina, S. (2021)]

[Firnkorn, D. et al. (2015); Yu, M. et al. (2018); Ma, D. et al. (2019); Chen, A. et al. (2022)].

**Concern # 6 - Comment #6-c.**

The introduction provides a good background of the problem and proposes a novel concept “the healthy core”, but it would benefit from more detailed explanation of why existing Domain Adaptation strategies are insufficient and how the "healthy core" concept improves upon them. Additionally, some technical terms (like "Domain Adaptation") are introduced without a clear definition, which might confuse readers not familiar with the terminology. Lastly, it would be beneficial to elaborate on the procedure of creating a "healthy core", even if it is explained later, to give the reader a clearer idea of what it entails.

**Response:** Thank you for the comment. We have revised the aforementioned sentences as follows:

However, most domain adaptation methods are designed to transform the entire dataset (including both HCs and patients) into a common space. While this approach is intuitive, it overlooks a potential concern: heterogeneity may exist even within HC cohorts. Consequently, transforming and aligning the entire dataset may not yield the expected effectiveness. To address this challenge, we propose a novel concept, the "healthy core," which involves transforming and aligning imaging data from HCs using their brain MRI data. This approach deviates from the traditional methodology by focusing specifically on HCs and their unique brain imaging characteristics.

**Concern # 7**

Methods: The methodology for data collection is clearly outlined, and the comprehensive nature of the data collected lends credibility to the research. However:

**Concern # 7 - Comment #7-a.**

Some more contexts could be provided to explain the relevance or importance of each specific type of data collected (e.g., BDI-II scores, STAI scores, etc.).

**Response:** Thank you for granting us the opportunity to provide additional details. While the current study did not specifically focus on assessing psychological factors of migraines, common tests such as the Beck Depression Inventory-II (BDI-II) and the State-Trait Anxiety Inventory (STAI) are often employed to evaluate symptoms of depression and anxiety.

We have incorporated the following paragraph into the methods section 2.3:

Data collected from all study participants encompassed age, sex, Beck Depression Inventory-II (BDI-II) scores, and State-Trait Anxiety Inventory (STAI) scores—commonly used measures for assessing symptoms of depression and anxiety. Additional data collected from migraine participants included headache frequency, number of years lived with headache, and Migraine Disability Assessment (MIDAS) scores.

**Concern # 7 - Comment #7-b.**

The list of exclusion criteria could be better structured to enhance readability.

**Response:** We have restructured the paragraph for better readability as follows:

Participants were recruited for the study between 2012 and 2021. All participants were male or female adults between the ages of 18-65. Those with contraindications to MRI, acute pain conditions other than migraine, neurological disorders other than migraine, women who were pregnant, and individuals with abnormal brain MRI findings according to usual clinical interpretation were excluded. HCs were excluded if they had a history of migraine, however, occasional tension-type headaches (< 3 tension-type headaches per month) were allowed.

**Concern # 7 - Comment #7-c.**

The participant selection criteria are well-detailed, providing a clear picture of the study population. One point might be useful to specify why the age range was specifically 18 to 65 years.

**Response:** Thank you very much for this comment.

In an effort to optimize enrollment success in our research studies, we selected to enroll participants ages 18 and 65. This age range aligns with the typical age demographics of migraine patients seen at Mayo Clinic.

**Concern # 7 - Comment #7-d.**

The paragraph “data collection” is well-structured and clear, but it might benefit from the use of sub-headings or bullet points for the details of each scanner's specifications to enhance readability. Authors may consider including this information in a table or in supplementary material.

**Response:** Thank you for the comment. We agree. We have re-structured the data collection section for better readability and included sub-headings and bullet points for scanner specifications.

Imaging Parameters:

Washington University: MAGNETOM Trio 3T scanner using a 12-channel head matrix coil.

- T1-weighted images: TE=3.16 ms, TR=2.4 s, 1x1x1 mm voxels, 256x256 mm field of view (FOV), acquisition matrix 256 x 256.
- T2-weighted images: TE=88 ms, TR=6280 ms, 1x1x4 mm voxels, 256x256 mm field of view, acquisition matrix 256 x 256.

Mayo Clinic: MAGNETOM Skyra 3T scanner using a 20-channel head matrix coil.

- T1-weighted images: TE=3.03 ms; TR=2.4 s; 1x1x1.25 mm voxels; 256x256 mm field of view, acquisition matrix 256 x 256.
- T2-weighted images: TE=84 ms; TR=6800 ms; 1x1x4 mm voxels; 256x256mm FOV, acquisition matrix 256 x 256).

**Concern # 7 - Comment #7-e.**

The relationship between DS1 and DS2 is unclear; it is uncertain whether they are two independent datasets or if DS2 is a subset of DS1, especially since both datasets contain patients scanned at the same center.

**Response**: Thank you for the comment. The DS1 and DS2 cohort were part of the same study. However, the DS1 cohort was enrolled at an earlier timepoint relative to the DS2 cohort. The DS1 cohort was enrolled first at Washington University and later at Mayo Clinic, whereas the DS2 cohort was enrolled only at Mayo Clinic.

**Concern # 7 - Comment #7-f.**

The sentence on page 6, "T1 images were used for ruling out gross structural abnormalities," could be rephrased as follows: "T1 images were employed to exclude any significant or clearly visible structural abnormalities”.

**Response:** Thank you for the comment. We have revised the aforementioned sentences as follows:

T1 images were employed to exclude any significant or clearly visible structural abnormalities.

**Concern # 7 - Comment #7-g.**

The sentence on page 6, “Data included in this analysis have been utilized in prior analyses [Schwedt, T.J., 2015 and Chong, C. D. 2017]”. Both datasets were utilized or one or subset of participants? It seems MRI T1-weighted images of 55 migraine patients and 58 healthy controls were used in that study, this is not the same number of participants in this current study. Please clarify.

**Response:** Thank you for allowing us to clarify. A subset of participants included in this study were included in prior publications.

We have revised the paragraph for better interpretability as follows:

Some of the participant data included in this study had been included in prior publications [Schwedt, T.J., 2015 and Chong, C. D. 2017].

**Concern # 7 - Comment #7-h.**

The sentence on page 7, “In order to avoid the confounding of multiple cohort differences, the mean and
variance differences were removed from each cohort to identify the remaining differences”:

i. Does the author intend to use “in order to avoid” or “in order to mitigate” in this context?
ii. The sentence could benefit from additional context and clarity. In statistics and data analysis, confounding often refers to the distortion of an estimated effect caused by an extraneous variable. In this case, it seems that the sentence is referring to the removal of mean and variance differences between cohorts to identify other (residual) differences more accurately. Please clarify.

**Response:** Thank you for the comment. You are right. We want to mitigate the potential interference from various cohort differences. Therefore, adjustments were made by eliminating mean and variance differences between cohorts to identify other (residual) differences more accurately. We have revised the aforementioned sentences as follows:

To mitigate the potential interference from various cohort differences, adjustments were made by eliminating mean and variance disparities within each cohort, enabling a more accurate identification of the residual differences.

**Concern # 7 - Comment #7-i.**

In the paragraph on page 8, the authors state, “We hypothesize that using a healthy core, a homogeneous subset of HCs from different sources, can help improve the generalization of the machine learning model for classifying migraine. This strategy addresses the heterogeneity of the HCs within and across the datasets and bridges the differences between the datasets.” This hypothesis should be introduced earlier in the manuscript, preferably in the abstract. This will give the reader a better opportunity to understand the manuscript and prepare for what is next.

The term 'hypothesis' is mentioned multiple times throughout the text; if there are multiple hypotheses, these should be presented earlier to familiarize the reader with the authors' intentions. It seems the authors might be confusing a hypothesis with a belief or expectation. A hypothesis is a testable proposition or prediction that is based on known information and that forms the basis for further investigation, while a belief is a personal view, opinion or conviction that does not necessarily have to be testable.

For instance, on page 7, the statement: “To capture the inherent variabilities, we hypothesize that a Geodesic Flow Kernel (GFK), which utilizes geometric manifolds termed Grassmann, may help," reads more like an assertion rather than a formal hypothesis. It could be rephrased as follows: "To capture the inherent variabilities, we utilized a Geodesic Flow Kernel (GFK) that employs geometric manifolds, termed Grassmann." Similarly, on page 4, the authors state: "We hypothesized that, compared to classification models developed without the use of a healthy core, imaging-based classification models for migraine developed with the use of a healthy core would have higher classification accuracy when applied to an unseen dataset." This also reads more like a belief rather than an established hypothesis. Please clarify.

**Response:** Thank you for the comment. We have revised the aforementioned sentences as follows:

To capture the inherent variabilities, we utilized a Geodesic Flow Kernel (GFK) that employs geometric manifolds, termed Grassmann.

We hypothesize that, compared to classification models developed without leveraging a healthy core, imaging-based classification models for migraine developed using a healthy core will achieve higher classification accuracy when applied to an unseen dataset.

**Concern # 7 - Comment #7-j.**

On page 8, the authors mention, "To achieve reliable results for the relatively small sample sizes, experiments are repeated under five different random scenarios (cross-validated) to build classifiers and report average performances." However, regardless of the number of times experiments are conducted, the small sample size presents a significant challenge in this study. This is a crucial limitation that needs to be clearly addressed in the limitations section, along with all the other concerns I have raised in my review. I would encourage the authors to further elaborate on these limitations in that section.

**Response:** Thank you for the comment.

**Concern # 8**

The results: Please address the following points:

**Concern # 8 - Comment #8-a.**

On page 9, I would recommend incorporating gender and age information (mean and standard deviation) into Tables 1 and 2 for improved clarity.

**Response:** Thank you for the comment. We have revised the aforementioned sentences as follows:

|  | HC  (Healthy Control) | EM  (Episodic Migraineurs) | CM  (Chronic Migraineurs) |
| --- | --- | --- | --- |
| DS1 (120 subjects) | 54  (Age: 30.17$\pm$10.97, F:39, M:15) | 51  (Age: 35.97$\pm$11.55, F:39, M:12) | 15  (Age: 34.75$\pm$11.00, F:13, M:2) |
| DS2 (76 subjects) | 42  (Age: 38.68$\pm$10.43, F:19, M:23) | 8  (Age: 39.62$\pm$10.97, F:2, M:6) | 26  (Age: 30.17$\pm$10.97, F:39, M:15) |

Regarding the size of the data, we have included it only in this response letter. However, if the reviewer prefers to have this information included in the manuscript, we are willing to include this.

|  | DS1 (66 migraineurs) | | DS2 (34 migraineurs) | |
| --- | --- | --- | --- | --- |
|  | EM | CM | EM | CM |
| Aura | 22  (Age: 35.97$\pm$11.55, F:14, M:8) | 6  (Age: 34.93$\pm$11.05, F:5, M:1) | 3  (Age: 40.15$\pm$10.46, F:0, M:3) | 15  (Age: 38.73$\pm$11.06, F:10, M:5) |
| No Aura | 29  (Age: 36.08$\pm$11.65, F:25, M:4) | 8  (Age: 36.60$\pm$11.00, F:7, M:1) | 5  (Age: 39.97$\pm$10.22, F:2, M:3) | 11  (Age: 37.90$\pm$9.34, F:10, M:1) |
| Unknown | 0 | 1  (Age: 32, F:1, M:0) | 0 | 0 |

**Concern # 8 - Comment #8-b.**

On page 10, I would suggest removing Table 3 and integrating the information directly into the text. This is because the table, as it currently stands, does not seem to add significant value to the content.

**Response:** Thank you for the comment. We removed Table 3.

**Concern # 8 - Comment #8-c.**

On page 10, the authors mention: “This result confirms that there are mean differences between the two cohorts for each modality.” Could it be that the authors meant “features” instead of “modality”? Please clarify.

**Response:** Thank you for the comment. The modality represents a group of features. For better clarity, we have revised the aforementioned sentences as follows:

This result confirms that there are mean differences between the two cohorts for each modality (mean, thickness, and volume features).

**Concern # 8 - Comment #8-d.**

Once again, on page 10, the authors state, “This result confirms that there are mean differences between the two cohorts for each modality” I have previously commented on this difference in the methods section and find it essential to emphasize my concern here, as the difference is perplexing. The authors interpret this difference as indicating that the healthy control groups are heterogeneous, which they suggest necessitates the establishment of a “healthy core.” However, there is no explanation as to why this large difference emerges between two groups of healthy controls with nearly identical age ranges (37 and 38 years old), scanned under nearly identical scanner settings, and whose data was processed using the same version of FreeSurferV5.3. An explanation for this difference is crucial. Is it artificially created and designed to facilitate the experiment? Could it be due to poor quality MRI images that required manual edits from different users? While differences between centers related to MRI processing are expected, the scale of the discrepancy observed here is unusually significant. Are these differences consistent with similar differences introduced in other multi center studies?

**Response:** Thank you for the comment. The significant difference observed was not artificially created; rather, it served as the motivation for this study. While many controllable factors were managed, substantial differences in the datasets emerged. We believe these differences may be inevitable in other multi-center studies.

**Concern # 8 - Comment #8-e.**

In Table 5, the header “DS1 vs DS2” is mentioned three times, which leads to confusion. Could you please clarify?

**Response:** Thank you for the comment. In order to avoid confusion, for better clarity, we have revised the aforementioned table as follows:

| Datasets | Training | Validation | Test |
| --- | --- | --- | --- |
| CM  DS1> DS2 | Samples from Dataset 1:  HC (n=43) vs. CM (n=12) | Samples from Dataset 1:  HC (n=11) vs. CM (n=3) | Samples from Dataset 2:  HC (n=42) vs. CM (n=26) |
| CM  DS2 > DS1 | Samples from Dataset 2:  HC (n=33) vs. CM (n=21) | Samples from Dataset 2:  HC (n=9) vs. CM (n=5) | Samples from Dataset 1:  HC (n=54) vs. CM (n=15) |
| EM  DS1 > DS2 | Samples from Dataset 1:  HC (n=43) vs. EM (n=41) | Samples from Dataset 1:  HC (n=11) vs. EM (n=10) | Samples from Dataset 2:  HC (n=42) vs. EM (n=8) |
| EM  DS2 > DS1 | Samples from Dataset 2:  HC (n=33) vs. EM (n=6) | Samples from Dataset 2:  HC (n=9) vs. EM (n=2) | Samples from Dataset 1:  HC (n=54) vs. EM (n=51) |

**Concern # 8 - Comment #8-f.**

On pages 17-18, the figures presented are simple scatter plots, the clarity of which could be improved. Also, please consider adding a legend to specify the meaning of the red and blue dots.

**Response:** Thank you for the suggestions. Each individual plot includes a legend describing the red and blue dots. The original legend size in our manuscript was small, so we have modified it accordingly in figure 3 and 4.

**Concern # 9**

Discussion and Conclusion:

**Concern # 9 - Comment #9-a.**

Please consider dividing the “discussion and conclusion” into two separate sections.

**Response:** Thank you for the comment. We have divided the 'discussion' and 'conclusion' into two separate sections.

**Concern # 9 - Comment #9-b.**

In the first paragraph of page 19, it's critical for the authors to define what they mean by “heterogeneous” with regard to the healthy control groups. This clarification is particularly important in providing a rationale for constructing the “healthy core.” The authors should elaborate on why their healthy control groups display significant differences in the mean values of features such as thickness, volume, and area. Without this explanation, it could be inferred that these differences were artificially manufactured to facilitate the experiment. While we expect differences in mean features among multi-center data, the large discrepancy observed in the authors' settings is quite puzzling.

**Response:** Thank you for the comment. For better clarity, we have revised the aforementioned first paragraph as follows:

The inherent differences in data characteristics stem from various factors, including variations in patient demographics and differences in data acquisition methods from multiple institutions or multi-center. The key discovery of this study lies in the effective utilization of a 'healthy core,' representing a uniform dataset sourced from a group of healthy individuals. This strategy significantly improves the performance of classification models for both EM and CM. Implementing a healthy core successfully counters the usual decline in model performance when applying the migraine classification model to distinct datasets gathered from patients across diverse institutions and scanned using varying devices. Depending on the modeling approach, integrating a healthy core led to classification accuracy peaking at 88% for both EM and CM.

**Concern # 10**

Limitations: While reviewing the manuscript, I found many limitations in this study. I encourage authors to think about the following points:

**Concern # 10 - Comment #10-a.**

Please consider creating a section with the header “Limitations”.

**Response:** Thank you for the comment. We have added a new section.

6. Limitations

Also, add several paragraphs as below.

Sample Size for Scalability: The study's statistical methods, MMD rely on sample sizes, denoted as 'm' and 'n,' from the distributions being compared. The quadratic dependence of MMD on sample size can pose computational challenges, particularly when dealing with substantial data volumes. While an adapted version of MMD with reduced computational cost exists, it is essential to recognize that in clinical studies with finite and manageable sample sizes, this limitation is less pronounced.

Interpretability of GFK Feature Space: The introduction of a new feature space by GFK that captures the gradual change from one dataset to another can be statistically meaningful. However, this feature space may not be directly interpretable in the context of the original data. Researchers should be aware that while GFK effectively identifies homogeneous characteristics, it is not designed for pinpointing specific differences between datasets within this novel feature space.

Data Quality: Both MMD and GFK are reliant on the quality of the data. Issues such as missing data, noise, and errors can impact the performance of these methods. Furthermore, data quality issues can affect the overall quality of the derived "healthy core." It is essential for researchers to ensure data quality and preprocessing measures to minimize the impact of these limitations.

**Concern # 10 - Comment #10-b.**

Consider the effect of sample sizes on the MMD calculation.

**Response:** Thank you for the comment. In practical applications, we acknowledge that MMD exhibits heightened sensitivity to distribution differences, particularly evident at small sample sizes, a common trait in statistical testing. However, within clinical domains, gathering a sizable dataset is often challenging. This challenge motivates us to aggregate data samples from various sources. We place our trust in the MMD test, which demonstrated commendable performance in experimental scenarios where the dimensionality significantly surpassed the sample size, typically fewer than 200. Despite conducting experiments multiple times to ensure reliable conclusions, the constraint of a small sample size remains a prominent hurdle in this study. This study is a proof of concept based on a dataset comprising 200 samples sourced from diverse institutions. Subsequent studies will further validate the migraine classification models presented here and explore the efficacy of incorporating a healthy core when constructing novel classification models.

**Concern # 10 - Comment #10-c.**

MMD relies on the assumption that the distributions being compared are homogeneous. If the data have underlying heterogeneities, MMD might not accurately represent the closeness of these distributions.

**Response:** Appreciation for your feedback. MMD represents a straightforward multivariate testing method used to compare two distributions, denoted as p and q. This involves utilizing samples of size m and n from the respective distributions. The test statistic, denoted as 'r,' signifies the discrepancy in mean function values between the two samples. A substantial 'r' suggests that the samples likely originate from distinct distributions. The term 'Maximum Mean Discrepancy (MMD)' refers to this test statistic. As per Gretton et al. in 2012, nonparametric statistical tests based on MMD are deemed distribution-free, indicating no assumptions are made about p and q. However, they tend to be conservative in detecting differences between the distributions. Our belief is that MMD remains applicable under the assumption of homogeneity in the distribution of the two datasets.

Gretton, Arthur, et al. "A kernel two-sample test." The Journal of Machine Learning Research 13.1 (2012): 723-773.

**Concern # 10 - Comment #10-d.**

The use of MMD and GFK can be computationally intensive, especially when dealing with large datasets. This could potentially limit the scalability of the proposed framework, particularly in real-world settings where datasets can be very large.

**Response:** Thank you for your feedback. We acknowledge the computational cost associated with calculating MMD, especially when dealing with substantial data volumes, due to its quadratic dependence on the sample size. To mitigate this limitation, an adapted version of the MMD statistic has been introduced, reducing the computational cost to linear with respect to the number of samples. Additionally, this modified version is accompanied by an asymptotic test, as outlined by Gretton et al. in 2012. This adaptation represents one approach to overcome the scalability challenge of the proposed framework. However, it's essential to clarify that this method primarily aims to tackle the inherent heterogeneity within a healthy control cohort and in multicenter studies. In a typical clinical study, the sample size is finite and manageable. When dealing with larger sample sizes, models can be constructed without the need for composite data from multiple sources. Therefore, our method remains highly advantageous in clinical studies — the harmonization method.

In order to acknowledge scalability limitation, we have stated the following paragraphs in the limitations section.

Sample Size for Scalability: The study's statistical methods, MMD rely on sample sizes, denoted as 'm' and 'n,' from the distributions being compared. The quadratic dependence of MMD on sample size can pose computational challenges, particularly when dealing with substantial data volumes. While an adapted version of MMD with reduced computational cost exists, it is essential to recognize that in clinical studies with finite and manageable sample sizes, this limitation is less pronounced.

Gretton, Arthur, et al. "A kernel two-sample test." The Journal of Machine Learning Research 13.1 (2012): 723-773.

**Concern # 10 - Comment #10-e.**

The GFK method is sensitive to the choice of parameters, such as the number of subspaces in the Grassmann manifold. Incorrect parameter selection can significantly impact the performance of the method.

**Response:** Appreciate your feedback. The GFK approach was introduced to simplify computations and reduce the requirement for tuning numerous parameters when compared to Gopalan et al.'s (2011) method. When aiming to pinpoint optimal subspaces within the Grassmann manifold, the choice of parameters holds great importance. The original GFK technique is straightforward to implement, requiring no cross-validation for subspace quantity (optimal number of subspaces). Leveraging the kernel trick, GFK can capture an infinite number of subspaces. Consequently, our proposed modification for the GFK component avoids the need for parameter selection concerning subspaces and kernels.

R. Gopalan, R. Li, and R. Chellappa. Domain adaptation for object recognition: An unsupervised approach. In Proc. of ICCV, pages 999–1006, 2011

Gong, B., Shi, Y., Sha, F., & Grauman, K. (2012, June). Geodesic flow kernel for unsupervised domain adaptation. In 2012 IEEE conference on computer vision and pattern recognition (pp. 2066-2073). IEEE.

**Concern # 10 - Comment #10-f.**

Another limitation to consider is that GFK creates a new feature space that captures the gradual change from one dataset to another. While this feature space can be statistically meaningful, it might not be directly interpretable in the context of the original data. This could pose challenges in understanding and interpreting the results.

**Response:** Thank you for your input. We fully agree with the reviewer’s viewpoint. While this feature space holds statistical significance, it may not offer direct interpretability within the original data context.

In geodesic flow kernel models, handling domain shift involves encompassing an infinite array of subspaces, capturing shifts in geometric and statistical properties from the source to the target domain. The strength of the GKF method lies in its ability to compare two datasets without requiring extensive cross-validation or labeled data from either domain.

However, we recognize that interpreting the outcomes can pose a challenge, as they do not explicitly disclose data differences within specific subspaces. However, in this study, our utilization of GFK does not aim to precisely identify differences between the two datasets within a novel feature space. Rather, our objective is to isolate the healthy core samples, highlighting their homogeneous characteristics amid the vast range of new feature representations.

In order to acknowledge Interpretability limitation, we have stated the following paragraphs in the limitations section.

Interpretability of GFK Feature Space: The introduction of a new feature space by GFK that captures the gradual change from one dataset to another can be statistically meaningful. However, this feature space may not be directly interpretable in the context of the original data. Researchers should be aware that while GFK effectively identifies homogeneous characteristics, it is not designed for pinpointing specific differences between datasets within this novel feature space.

**Concern # 10 - Comment #10-g.**

Both MMD and GFK are dependent on the quality of the data. Issues like missing data, noise, and errors can impact their performance. Furthermore, these issues can affect the quality of the derived “healthy core.”

**Response:** Thank you for the comment. We state this in the limitation section.

Data Quality: Both MMD and GFK are reliant on the quality of the data. Issues such as missing data, noise, and errors can impact the performance of these methods. Furthermore, data quality issues can affect the overall quality of the derived "healthy core." It is essential for researchers to ensure data quality and preprocessing measures to minimize the impact of these limitations.

**Comments from Reviewer #2:**

Congratulations on your interesting work, which aims to tackle the challenge of having generalizable neuroimaging classification models and end up proposition a "healthy core" in the lines of "normative models", being taken by other researchers.

I believe that in the current form, the manuscript is not delivering its suitable potential, not only in clinical or physiological interpretability (the most important) and also regarding reproducibility of your work or methods by others. The materials and methods section feels incomplete or unclear, and some snipets of text seem to be misplaced in the results section. Also regarding results, be aware of the interpretation of machine-learning results, and notably the influence of having imbalanced datasets and the interpretation you derive from ROC AUC values. Maybe consider having a stronger support of someone more seasoned on those topics.

Also, I'm sending you in the attachment the pdf manuscript with suggestions of edits, comments and questions.

Please address these issues and provide a more clinical interpretation of the features chosen by the algorithms and models derived from such, to elevate your paper.

Consequently, I feel that your paper needs a major revision before being ready for publication and I'll be available to help with the review of the improved version.

**Comments on # Page 3**

should use ; instead of , to separate references ...

uniformize: some publication years with parentheses others without

also, so authors inicials have . others do not

**Response:** Thank you for the comment. We have revised the aforementioned sentence as follows:

[Eck, J. et al. (2011); Lo Buono et al. (2017); Meylakh, N. et al. (2018); Mu, J. et al. (2020); Wei H. et al. (2020); Scheepens, D. S. (2020); Ashina, S. (2021)].

**Comments on # Page 4**

some authors do not have initials ...

s

**Response:** Thank you for the comment.

[Tzeng, E. et al. 2017, Csurka, G. et al. 2017, Tang, H. et al. 2020].

[Yao, Y. et al. 2010, Gong, B. 2012, Matasci, G. 2015, Tzeng, E. et al. 2017, Csurka, G. et al. 2017, Tang, et al. 2020].

**Comments on # Page 5**

why the different font size?

years old

what about psychiatric disorders?

extra space

.

all Siemens scanners have been called Magnetom something

Skyra

20

12

**Response:** Thank you for the comment. We have revised the aforementioned sentence as follows:

Participants were recruited for the study between 2012 and 2021. All participants were male or female adults between the ages of 18-65 years old. Those with contraindications to MRI, acute pain conditions other than migraine, neurological disorders other than migraine, women who were pregnant, and individuals with abnormal brain MRI findings according to usual clinical interpretation were excluded. HCs were excluded if they had a history of migraine, however, occasional tension-type headaches (< 3 tension-type headaches per month) were allowed.

Study participants were imaged on one of two Siemens (Erlangen, Germany) scanners, each at a different institution. Scanners at both institutions differed in scanner model (MAGNETOM Trio vs MAGNETOM Skyra),

However, we have not tested whether psychiatric disorders significantly affect the outcomes. This remains a topic for future work.

**Comments on # Page 6**

FOV is an area

define acronyms

voxels are imaging volumes!

2

3

3

hyphenate field-of-view

what sequences were used? 2D or 3D? If 3D, MPRAGE for T1w, SPACE for T2?

did you use T2w or T2 FLAIR weighted? From the parameters alone I would say you have used a 3D T1w sequence, namely MPRAGE. Both MPRAGE and FLAIR sequences use an inversion time TI! And what were the nutation/flip angles?

use a space between number and unit

remove parenthesis

Question: why the different parameters. This being said, some of the parameters, e.g TE, are not significantly different

as far as image constrast is concerned!

If so, I would imagine that you have used 3D T1w MPRAGE sequences

space needed

you have not used subcortical volumes from the DSK atlas?

**Response:** Thank you for the comment.

**Comments on # Page 7**

**Question:** were variable distributions normal? if so state the testing and results.

**Response:** We examine both parametric tests, assuming normality, and nonparametric tests, which do not rely on any distributional assumptions.

**Question:** which significance level was used? 5%?

**Response:** Thank you for the comment. Yes, we used the 5% significance level. In order to clarify this, we have revised the aforementioned sentence as follows:

The cohort-wise means/variances of each feature were compared using a two-sample t-test/F-test under the 5% significance level. False Discovery Rate (FDR) under the 5% significance level was used to correct for multiple comparisons.

**Question:** Did you use any particular software for conducting the statistical analysis?

**Response:** Yes, we used MATLAB and python for conducting statistical tests and applying the proposed algorithms.

**Question:** I believe details are missing regarding the methods for constructing the healthy core.

**Response:** The entire process of constructing the Healthy Core is depicted in the visual representation presented in Figure 1. The proposed model, comprising the unified GFK+MMD framework, is provided in detail within the appendix.

**Question:** How did you do it in practice, how could the reader reproduce your approach?

**Response:** We can share the code upon request.

**Comments on # Page 8**

**Question:** I would suggest including a subsection just for the classification tasks. Need to provide more details on the classification tasks. sizes of training, validation and test sets.

**Response:** Thank you for the comment. Subsection 3.2 to 3.4 covers the details of classification tasks. Also, the size of training, validation, and test sets are in the table 5, 7, and 9.

- Experiment I: evaluating the performance of different classification models within a single dataset
- Experiment II: evaluating the performance of the classification models developed on one dataset and tested on a second dataset
- Experiment III: evaluating the performance of classification models with and without using Healthy Core

**Question:** were cross-validations done using randomly selected cases or every case was featured both in the training and

validation set across folds?

**Response:** In the training of the classification models, cross-validation has been applied. To get reliable results for the relatively small sample sizes, experiments are repeated under five different random scenarios (cross-validated) to build classifiers and report average performances. Since our last experiment focuses on migraine only, we report the Area Under the Curve (AUC) for the first two experiments and the accuracy of identifying migraine in the last experiment.

**Question:** were training, validation and test sets balanced for age, sex, scanner parameters,

pathology, etc? If so, what was the approach?

**Response:** In the case of a small sample size (< 200), it's not feasible to create balanced training, validation, and test sets based on age and sex. Instead, we employed cross-validation without stratification, which we believe is a more practical approach in a clinical setting. Our analysis revealed that age does not significantly differ across datasets. Regarding sex differences, as previously mentioned in the manuscript, we thoroughly examined the impact of sex differences on dataset variations. Based on the results of our experiments, we have confirmed that differences in sex distribution may not significantly affect the classification results.

We randomly selected the sex-balanced sub samples from both cohorts. After making the balanced groups, the mean and variance of features (volume, area, and thickness) were tested using the t-test and KS test for mean, F-test and Ansari-Bradley test for variance whether the dataset difference exists. Since we selected a random subset to test, this test procedure was repeated 10,000 times to avoid a sampling-dependent conclusion. It was concluded that there is a significant mean difference and a marginal variance difference in each feature (volume, area, and thickness), but sex distribution was not a significant factor in the dataset difference.

**Question:** was there any data augmentation?

**Response:** No, we did not employ any data augmentation. Instead, the use of GFK allowed us to explore various data distributions across the manifold.

**Question:** what were the algorithms used for classification?

**Response:** We employed four binary classifiers: regularized logistic regression, support vector machine (SVM), random forest, and XGBoost. The descriptions are in the experiment section.

**Question:** Did you do hyperparameter fine-tuning? Any nesting?

**Response:** For classification models, we applied the Bayesian optimization to efficiently get the best hyperparameters for each model. For GFK-MMD, our proposed modification for the GFK component avoids the need for parameter selection concerning subspaces and kernels.

**Question:** You only used AUC and accuracy as figure of merits for the performance of your models?

**Response:** While accuracy is a useful metric for evaluating performance under a specific threshold for class determination, it may not be sufficient when dealing with imbalanced cases. In such scenarios, the Area Under the Curve (AUC) is more suitable for assessing performance. Although there are other metrics, such as F1-score, precision, recall, and more, comparing the AUC and accuracy when evaluating different methods for classification is appropriate.

**Response:** Thank you for the comment.

**Comments on # Page 9**

mean+-standard deviation, if so need to mentioned that

age range?

51+15?

years old

were patient and HC groups age and sex matched?

space

is not one decimal place enough from the clinical point-of-view?

. The

years old; age range?

one decimal place

**Question:** should include the definition of acronyms either in the table caption or just below the table. Tables and figures should be

on itself, and as reasonable as possible, self-understandable without resorting to the main text.

**Response:**

|  | HC  (Healthy Control) | EM  (Episodic Migraineurs) | CM  (Chronic Migraineurs) |
| --- | --- | --- | --- |
| DS1 (120 subjects)  Dataset 1 | 54  (Age: 30.17$\pm$10.97, F:39, M:15) | 51  (Age: 35.97$\pm$11.55, F:39, M:12) | 15  (Age: 34.75$\pm$11.00, F:13, M:2) |
| DS2 (76 subjects)  Dataset 2 | 42  (Age: 38.68$\pm$10.43, F:19, M:23) | 8  (Age: 39.62$\pm$10.97, F:2, M:6) | 26  (Age: 30.17$\pm$10.97, F:39, M:15) |

**Question:** this is a parameter that was not mentioned in the methods section.

**Response:** This does not pertain to the parameter that represents the quantity of subjects in the healthy control group, episodic migraineurs, and chronic migraineurs within two distinct datasets.

**Question:** of which brain regions? I would strongly suggest including supplementary data. which ones 41? and which 3? showing this.

this is not the difference, right? this shows the number of significantly different features ...

need to show which features are these ...

**Response:** Section 3.1 is not intended to present a primary discovery. Its primary objective is to evaluate the distinctions in features between the two healthy control datasets, emphasizing the importance of these variances. In this section, it is essential to establish the motivation rather than providing a detailed breakdown of the 41 and 3 features individually. Our perspective is that identifying specific brain regions where differences occur in healthy controls is not the primary focus.

**Comments on # Page 10**

**Question:** too small lettering. Cannot read properly.

**Response:** Thank you for your comment. We have made updates to the figure and its corresponding description.

Area Features	Thickness Features

Volume Features

Figure 2. Univariate Analysis for Mean Difference Scale

Note: $\mu_{DS2HC}$ represents the Feature Average of Healthy controls in Dataset 2 and $\mu_{\mathrm{DS}1\mathrm{HC}}$ depicts the Feature Average of Healthy controls in Dataset 1

**Question:** this means that there are differences regarding sex, or that your significance level considered is 1%?

**Response:** Appreciate your feedback. At this stage, it does not directly suggest differences related to sex. Our goal is to investigate whether the significant variations in sex distribution within groups could potentially contribute to the differences observed within the cohort. We have chosen a p-value=0.01 to adopt a more conservative approach, strengthening our ability to detect this pattern across multiple randomized tests.

**Comments on # Page 11**

**Question:** what does this mean in practice? how did you balance the samples?

for which brain region? are these overall values averaged for all brain regions? If so, why?

what does this mean, balanced how?

**Response:** In the case of two cohorts, statistical tests can determine if there are significant differences between the two groups. However, when multiple factors are involved, arriving at a definitive conclusion becomes more complex.

In this subsection (3.1), it is worth noting that the mean and variance of features between the two groups, both of which consist of healthy control data, show significant differences so far. The purpose of this investigation, conducted through random sampling, is to explore the possibility that these significant group differences may stem from disparities in sex distribution.

By randomly selecting samples for each group and ensuring sex balance in subgroups, we can evaluate the differences in mean and variance. To mitigate the potential bias stemming from sampling, we repeat this procedure multiple times (100 repetitions).

**Question:** this should be stated in the methods section. did you do similarly for the remaining statistical tests?

**Response:** Section 3.1 is not intended to present a primary discovery. Its primary objective is to evaluate the distinctions in features between the two datasets, emphasizing the importance of these variances. No, this comparison is specific to this section. Subsequent comparisons in the following experiments are based on model performances.

**Question:** define acronym.

**Response:** Thank you for the comment. We have revised the aforementioned sentence as follows:

After making the balanced groups, the mean and variance of features (volume, area, and thickness) were tested using the t-test and the Kolmogorov-Smirnov (KS) test for mean, F-test and Ansari-Bradley test for variance

**Question:** you are accounting here both HCs and patients, right?

**Response:** No, in subsection 3.1, our goal is not to differentiate between healthy controls and patients. Instead, this subsection is dedicated to exploring disparities among healthy controls. This exploration serves as motivation for the development of our healthy core algorithm, which will aid in distinguishing between the healthy core and patients in the subsequent section.

**Question:** do not understand? where do you have the number of features?

what are the dispersion metrics of such differences ? (e.g. standard deviation)

**Response:** Examining 204 features in every single experiment can be challenging. Hence, we compare the average 'mean' difference across all 204 features. The numbers in the table represent the cumulative differences over all features.

Thank you for the comment. We have revised the aforementioned sentence as follows:

Table 4 provides an overview of the average differences in means across various scenarios, including DS1 vs DS1, DS2 vs DS2, random splits, and DS1 vs DS2. In the case of DS1 vs DS1, we examined 21 samples randomly selected from the DS1 dataset and another 21 non-overlapping samples from the same dataset. It is important to note that in Dataset 2, there are a total of 42 Healthy controls, which was evenly divided between the two datasets. We ensured that each subgroup had a maximum of 21 samples, chosen carefully for consistency and balance across all four scenarios. For the 'random splits' scenario, we randomly select 21 subsets from the combined dataset (DS1 and DS2) and included another 21 non-overlapping samples. The numbers presented in the table represent the mean features for each subset and the variations between them. Given the inherent randomness in the sampling process, we repeated this procedure 100 times and reported the averaged results.

**Question:** what are the dispersion metrics of such differences ? (e.g. standard deviation)

**Response:** The standard deviation of mean differences in features provides a consistent outcome compared to the average mean difference. However, we have chosen not to include this table in the manuscript.

| $\sigma(\mathrm{Av}g. \vert DS1-DS2\vert)$ | DS1 vs DS1 | DS2 vs DS2 | Random splits | DS1 vs DS2 |
| --- | --- | --- | --- | --- |
| Area (mm^2^) | 13.23 | 12.72 | 13.19 | 18.97 |
| Thickness (mm) | 0.00824 | 0.00749 | 0.00763 | 0.00881 |
| Volume (mm^3^) | 31.47 | 30.29 | 31.32 | 32.65 |

**Question:** why do you say that? What is the criteria for compatibility?

**Response:** In all possible comparisons in the table 4, the average mean difference within a dataset is smaller compared to that between datasets. Furthermore, we conducted a t-test on the 204 difference values between the two groups, and the results confirm that DS1 and DS2 are not statistically compatible at the 5% significance level.

**Comments on # Page 12**

**Question:** why the choice of these ones?

**Response****:** We chose well-established machine learning algorithms for binary classification. Given the sample size of our clinical experiments, we prioritized the robustness of these four algorithms (regularized logistic regression, support vector machine (SVM), random forest, and XGBoost) in handling hyperparameter selection. While we present results using these four classification models, it is also possible to explore the extension of other algorithms.

**Question:** but the folds include only the training and validation sets, right? with 5 folds, is that it?

again, always define the acronyms and abbreviations used in the table ...

I suggest including a schematic in the materials and methods section about how you did the machine learning ...

use a more informative title.

you have quite

small datasets.

**Question:**

accuracy chance level 11/(11+3) ~79%

accuracy chance level 11/(11+3) ~73%

accuracy chance level 11/(11+10) ~50%

accuracy chance level 8/(8+2) ~80%

Why did you consider only AUC as a model performance metric? This is not the best metric, especially in

imbalanced datasets such as as yours. What are the chance values for the metrics?

what features were used for classification? all volumes, areas and thicknesses for all brain regions, or did you do any prior feature selection?

maybe below chance level below chance

**Response:** Accuracy and AUC (Area Under the Curve) are both metrics used to evaluate the performance of classification models, such as in machine learning or medical diagnosis. However, they capture different aspects of classification performance, and the choice between them depends on the specific characteristics of the problem at hand.

**Accuracy** measures the ratio of correctly classified instances to the total number of instances. While accuracy is a commonly used metric, it can be misleading in situations with imbalanced datasets. For example, in a medical diagnosis task where only a small fraction of patients have a rare disease, a model that classifies all instances as non-diseased can still achieve high accuracy, even though it's practically.

**AUC (Area Under the Curve)**, on the other hand, evaluates a model's ability to distinguish between positive and negative classes across different probability thresholds. It is particularly useful when dealing with imbalanced datasets because it considers both true positive rates and false positive rates at various threshold levels. The AUC score summarizes the overall discriminative power of a model, providing a more comprehensive view of its performance.

AUC is often preferred when dealing with imbalanced datasets or when the focus is on the model's ability to rank instances correctly rather than classifying them with strict accuracy. It is a more robust metric for evaluating classification models in situations where class distribution is skewed or when the costs of false positives and false negatives are uneven.

**Comments on # Page 13**

**Questions:** where are these algorithms coming from? did you coded yourselves? also, did you do any parameter optimization?

**Response:** We can share the code upon request. For classification models, we applied the Bayesian optimization to efficiently get the best hyperparameters for each model. For GFK-MMD, our proposed modification for the GFK component avoids the need for parameter selection concerning subspaces and kernels.

**Questions:** the chance accuracy level for CM and DS1 is 11/(11+3) or ~79%. whilst this is not AUC this shows that your models should outperform this level. why is it satisfactory? any model and with AUC <0.700 is hardly useful for clinical use

**Response:** AUC is not the same as accuracy. While the model's average AUC performance was 0.6965, we achieved an accuracy higher than 0.88 by carefully selecting the algorithm threshold for class determination. We acknowledge the reviewer's suggestion and have removed the sentence 'The model performance may still be considered satisfactory' from the manuscript, as pointed out by the reviewer.

The only model with performance above chance would seem to be DS1 for EM, with AUC values maybe nearing clinical relevance

Notice this model corresponds to the largest sample set and with balanced groups!

chance level of 42/(42+26) ~62%

accuracy

**Comments on # Page 14**

**Questions:** chance level 54/(54+15) ~78%

chance level 42/(42+8) ~84%

chance level ~51.4%

Should also consider accuracy and other additional metrics

below chance level

should be below chance level

maybe slightly above chance

below chance

**Response:** Thank you for your suggestion. As mentioned on the previous page, it's important to distinguish between AUC and accuracy. While the model's average AUC performance was 0.6965, we achieved an accuracy exceeding 0.88. However, it's essential to note that accuracy may not be a suitable metric for small sample sizes and imbalanced datasets. This is why we have presented AUC in this table. It's not uncommon to observe lower performance in cross-domain experiments when heterogeneity exists between the training and test datasets.

**Comments on # Page 15**

I would say more relevant than that is the small sized datasets and the imbalanced groups

what is the composition of the healthy core? any particular subjects? any particular choice of features?

small test set

very small test set

You need to be much more detailed on the results regarding constructing the healthy core ...

**Response:** Thank you for your suggestion. The entire process of constructing the Healthy Core is depicted in the visual representation presented in Figure 1. The proposed model, comprising the unified GFK+MMD framework, is provided in detail within the appendix. Also, we mentioned in the following rebuttal.

**Comments on # Page 16**

**Questions:** are these values means across folds? if so please state that and the dispersion (e.g. standard deviation, std) define acronyms are these average values across the different algorithms? if so please state that.

**Response:** We have introduced acronyms for keywords in the manuscript when they are used for the first time. However, for frequently repeated keywords, we have retained the acronyms to maintain conciseness in the manuscript.

**Questions:** are these average values across the different algorithms? “Without using the healthy core, the four trained classification models do not have generalization accuracy to classify CM”

**Response:** No, we displayed the classification performance for each model individually. It is important to clarify that generalization accuracy in the manuscript, often referred to as test accuracy or out-of-sample accuracy, is a metric used in the evaluation of machine learning models, particularly in the context of classification tasks. It assesses the model's performance on a dataset it has never encountered before, helping us understand how well the model can generalize its predictions to new, unseen data.

**Questions:** how many HCs from each dataset comprise the healthy core

**Response:** The detailed sample number of healthy core is in the Table 10. We identified 28 healthy core in this study.

**Questions:** why not present the healthy core before the classification results?

**Response:** The table 10 shows classification accuracy of four models with and without Healthy Core for CM and EM Classification.

also consider including an extra column in table 11 with such mean+-std values

what were the MMD values and any eventual GFK metric?

in this manner you could follow a more consistent narrative, just as you present it in the

material and methods section.

**Comments on # Page 17**

**Questions:** I would say fist and second dimensions for the different plots represent different things, right?

actually they look the same for A and C and for B and D. Please discuss this.

**Response:** We present t-SNE plots of DS1 and DS2 for Chronic Migraine (CM) on the first and second principal components: (A) combines Healthy Controls (HCs, n=96) vs. CM from DS1 (n=15) and (C) combines HCs (n=96) vs. CM from DS2 (n=26). Upon close examination of the figures, it becomes evident that the healthy core is located in similar positions, while CM from Dataset 1 in (A) and CM from Dataset 2 exhibit differences. Similarly, figures (B) and (D) depict Healthy Core vs. CM1 and CM2. Although the figures are not identical, they exhibit similarities for better comparison.

**Questions:** why the dispersion of patient-related points, are these encompassing patient variability? would you expect and pattern also for patients?

use small cap t

**Questions:** which HCs points correspond to DS1 and to DS2, is there any pattern? Maybe I would expect that given the differences you described in section 3.1

**Response:** In Section 3.1, we specifically examined differences among healthy controls. You are correct in noting that the left-hand-side figure includes all healthy controls, while the right-hand-side figure focuses solely on the healthy core. Our objective is to accurately differentiate between healthy individuals and patients. However, there was significant heterogeneity among healthy controls, making it challenging to discern whether a subject is a Migraineur or not. This challenge highlights the importance of the healthy core as a more reliable reference for distinguishing patients from healthy individuals.

**Questions:** what encompasses the different dimensions? in particular the second dimension in B and D seems to be able to scatter patient-related points from a scale between -2 to 2 for A and C, to -50 to 60!

**Response:** The scale is not a critical factor. PCA or t-SNE captures lower-dimensional features from the original high-dimensional data, and these scales can vary depending on the samples used. It is important to focus on the relative scales across samples rather than absolute values. For example, you can observe how the scales of Principal Components (PCs) change depending on the training samples.

**Comments on # Page 18**

**Questions:** is why pattern from B so different from D and from B and D from fig. 3?

**Response:** Episodic migraineurs exhibit characteristics that are relatively close to the healthy core when compared to chronic migraineurs. It's important to note that in (B, D) of Figure 3 and (B, D) of Figure 4, their scales differ. Please consider whether the healthy core effectively distinguishes itself from the patients, as this is a crucial aspect of the figure.

**Questions:** why do you call it noise?

**Questions:** are the features and dimensions the same for figures 3 and 4?

**Response:** Yes, we use 204 features but they are embedding to the lower dimensional space. For visualization, the first two dimensions are used. Each dimension is from all 204 features (volume, area, and thickness). t-SNE is a common visualization technique in the machine learning community. t-SNE, which stands for t-Distributed Stochastic Neighbor Embedding, is a dimensionality reduction technique used in machine learning and data visualization. It is particularly useful for visualizing high-dimensional data in a lower-dimensional space while preserving the structure and relationships between data points. t-SNE was developed by Laurens van der Maaten and Geoffrey Hinton in 2008.

**Questions:** need to deepen the results presented, what are the main features that translate the HC group?

need to provide some ground basis for clinical/neurological/physiological interpretability ...

**Response:** The identification of the healthy core using our proposed method, which leverages the Geodesic Flow Kernel (GFK) and Maximum Mean Discrepancy (MMD), reveals **a higher level of homogeneity**. We observe that the descriptive statistics of brain regions within this identified healthy core exhibit remarkable similarity when compared to the descriptive statistics of brain regions within the composite of all healthy cores.

**This similarity highlights consistent patterns among healthy cores derived from multiple subjects, all recruited under the same protocol**. **These findings provide a robust basis for distinguishing subtle changes that occur in individuals with migraines.** In essence, our approach reveals common features and characteristics in healthy subjects that can serve as a benchmark for understanding and detecting deviations associated with migraine pathology, enhancing the clinical, neurological, and physiological interpretability of our results.

It is worth noting that further interpretations of these results will be pursued in our future work.

**Comments on # Page 19**

**Questions:** and therefore failing when it would be used in a completely ..

**Response:** Thank you for the comment. We have revised the aforementioned sentence as follows:

Although this type of approach is valid and well-accepted, and therefore failing when it would be used in a completely, it likely leads to overestimating the performance of the model

**Questions:** Maybe unnecessary statement or rephrase it ... it is not a more conservative estimate, not using

independent dataset would result in hyped estimates ... maybe use considered a more robust

**Response:** Thank you for the comment. We have revised the aforementioned sentence as follows:

Given that separate training and testing datasets were employed in the conducted experiments, it is advisable to view the reported classification accuracies as more cautious approximations of the model's performance.

**Comments on # Page 20**

**Questions:** need to describe much better. also, will the code be made available?

**Response:** Thank you for the comment. We have revised the aforementioned sentence as follows:

The code for the proposed healthy core algorithm is readily available and can be shared upon request.

**Comments on # Page 21**

**Questions:** “Future studies should more deeply phenotype the healthy control subjects in search of sources of heterogeneity that might associate with differences in brain structure

“

well but you can already do that with descriptive statistics of brain regions volumes, areas and thicknesses! much simpler than doing ML models!

this should already be done for this paper! use metrics such as mean, std, coefficient of variation, medians, IQR, even skewness and kurtosis

and even boxplots or rather violin plots ... this is actually what you should do before running any ML model, statistics of your variables!

**Response:** Thank you for your comments. While we have not included additional metrics in the manuscript, we've already conducted extensive comparisons involving 1st, 2nd, and higher-order statistics, as well as MMD, based on descriptive statistics of brain regions. Section 3.1 provides a comprehensive description of our exploration efforts. Despite addressing some distribution shifts, heterogeneity still persists, which has driven our motivation to develop a harmonized healthy core for better decision-making. However, the limitation we mentioned does not pertain to repeating experiments based on descriptive statistics. Instead, we acknowledge the need for further extensions to explore and identify sources of heterogeneity that may be associated with differences in brain structure.

**Questions:** need to review and complete the references. any page numbers were the specific content may be found?

**Response:** Thank you for the comment. We have revised the aforementioned sentence as follows:

Sun B., Feng J., & Saenko K. Correlation alignment for unsupervised domain adaptation. Domain adaptation in computer vision applications. 2017; 153-171.

**Questions:** Is this a book? an article?

**Response:** Hutson, M, Artificial intelligence faces reproducibility crisis. SCIENCE. 2018; 359(6377): 725-726

**Questions:** what does it mean the acronym?

**Response:** Thank you for the comment. We have revised the aforementioned sentence as follows:

Long M, Cao Y, Wang J, & Jordan M, Learning transferable features with deep adaptation networks. In International conference on machine learning. 2015, June; 97-105. PMLR (Proceedings of Machine Learning Research).

**Comments on # Page 25**

**Questions:** The appendix should be mentioned in the main text.

**Response:** Thank you for the comment. We have revised the aforementioned sentence as follows:

The entire process of constructing the Healthy Core is depicted in the visual representation presented in Figure 1. The proposed model, comprising the unified GFK+MMD framework, is provided in detail within the appendix.

**Questions:** what does sup mean?

**Response:** In mathematics, the term "supreme" (often denoted as "sup") stands for the supremum of a set of numbers. The supremum is a concept similar to the maximum but with a subtle difference. The supremum of a set is the smallest number that is greater than or equal to all the numbers in the set, but it doesn't have to be an actual member of the set. In simpler terms, it's the lowest upper bound. For example, if you have a set of numbers, the supremum is the smallest number that is still greater than or equal to every number in that set, even if it's not actually in the set itself.

**Questions:** explain how GFK is used here. what does G mean?

**Response:** The Geodesic Flow Kernel (GFK) facilitates the comparison of datasets by analyzing the interconnections among data points, taking into consideration the inherent shape and structure of the data. It serves as a method for quantifying the similarity between datasets based on their underlying structures. When provided with two subsets from distinct datasets, we can apply various algorithms to explore the structural similarities from multiple perspectives, including mean differences, variance differences, kurtosis differences, and more. Notably, GFK doesn't depend on hyperparameters, making it an efficient tool for swiftly comparing datasets using the kernel trick.

GFK explores multiple feature representations between two datasets and generates a range of transformations that describe changes in geometric and statistical properties as we transition from one dataset to another. This approach investigates various statistically significant feature representations between datasets, offering the advantage of capturing data variabilities rather than relying solely on a single snapshot of each dataset. We employ the Maximum Mean Discrepancy (MMD) as a tool to compare these feature representations.

**Questions:** what does G mean?

**Response:** In the context of machine learning, "GFK" typically stands for "Geodesic Flow Kernel." The "G" in GFK does not have a specific meaning; it's simply an abbreviation for the full term "Geodesic Flow Kernel." The Geodesic Flow Kernel is a mathematical method used to measure the similarity between two datasets by considering the underlying structure and shape of the data, but the "G" itself does not convey any particular meaning. In this context, G stands for GFK. We have revised the aforementioned sentence as follows:

// $G$ is a positive semidefinite matrix, geodesic flow kernel (GFK)
