# Supplementary material for: Healthy core: Harmonizing brain MRI for supporting multicenter migraine classification studies
Source: PLoS One. 2024 Dec 31;19(12):e0288300. doi: 10.1371/journal.pone.0288300 (PMC11687649; doi:10.1371/journal.pone.0288300)
Supplement: Attachment — Submitted filename: Response letter to PlosOne_R3_Final.docx [file pone.0288300.s006.docx]

We want to thank to the editor and the reviewer for the insightful review. We have carefully addressed all the comments and suggestions provided by the reviewer and believe that these revisions have significantly improved the quality and clarity of our manuscript.

In this submission, we have included a detailed point-by-point response to each comment raised by the reviewer along with the corresponding changes highlighted in the revised manuscript.

We believe that our proposed 'healthy core' methodology offers a novel and robust approach to harmonizing multicenter brain MRI data and improving the generalizability and accuracy of migraine classification models. We hope that the reviewers and readers will find our contributions valuable.

**Comments from Reviewer #4:**

**Question #1.**

The discussion lacks a comparison with previous works and references. The only sentence where works are mentioned is 'Many prior publications (including some of our own) reported the accuracy of classification models for migraine train and test using the same dataset, with strategies such as the "leave-one-out" methodology [Schwedt, T.J. et al. 2015, Chong, C.D. et al., 2017, Lee M.J. et al., 2019, Zhang, Q. et al., 2018, Yang, H. et al., 2018, Chong, C.D., 2021].' It is not possible to evaluate the goodness of the method if not compared to other models, even those not on migraines.

**Response:** Thank you for highlighting the need for a more thorough comparison with previous works and references. We agree that such comparisons are crucial for evaluating the robustness of our method. Our proposed method 'healthy core' represents the first attempt to harmonize multicenter brain MRI data by identifying a homogeneous subset of healthy controls, which can then be used as a reference prototype to minimize the differences between datasets. This innovative approach contrasts with traditional harmonization techniques that primarily focus on transforming the data to adapt to different datasets.

While it may appear that we have not directly compared our method with existing harmonization techniques, it is important to note that traditional methods like ComBat harmonization and other statistical or deep learning-based approaches transform the entire dataset to achieve adaptation. In contrast, our method leverages Maximum Mean Discrepancy (MMD) and Geodesic Flow Kernel (GFK) to create a 'healthy core' prototype, which serves as a reference to reduce variability across datasets. This fundamental difference in methodology makes direct comparison challenging but highlights the unique advantages of our approach.

We have demonstrated the effectiveness of the 'healthy core' concept through extensive experiments involving four different machine learning algorithms—Regularized Logistic Regression, SVM, Random Forest, and XGBoost. These experiments show the performance improvements when using the 'healthy core' for external validation on new cohorts of Episodic Migraine (EM) and Chronic Migraine (CM) patients. Specifically, we provided evidence that incorporating the 'healthy core' significantly enhances the model's accuracy and generalizability compared to not using it.

Our results are promising and indicate the potential of the 'healthy core' methodology to be combined with various machine learning and deep learning techniques. This adaptability further underscores the robustness and applicability of our approach across different scenarios and datasets.

We hope the reviewer will agree with us and consider our rationale is justifiable and satisfactory to address this comment.

**Question #2.**

There are too many tables and they are not well structured:

**Response:** Thank you for your suggestions. We have made the following changes to improve the structure and readability of the tables:

Combined Table 1 and Table 2: We have merged Table 1 and Table 2 into a single, more structured table. The revised table now includes age and gender variables in separate rows. This restructuring improves readability and provides a clearer overview of the participant demographics and clinical characteristics.

Combined Table 6, 8, and 10: Tables 6, 8, and 10 have been combined into a single table, with rows indicating the specific experiment they are linked to (Experiment 1, 2, or 3). This consolidation helps streamline the presentation of the experimental design and results, making it easier for readers to follow the study progression.

Revised Table Captions: We have revised all table captions to be more explanatory. The symbols and arrows have been clearly defined in the captions, explaining their meaning in the context of the experiments. For example, "DS1 → DS2" indicates that training was conducted on DS1 and testing on DS2.

Table 1. Demographics and clinical characteristics of study participants, including healthy controls (HC), episodic migraine (EM), and chronic migraine (CM) patients from two datasets (DS1 and DS2). The table includes sample size, mean age with standard deviation, gender distribution (female/male), and the presence of aura.

Table 5. Data split design for Experiments I, II, III Overview of training, validation, and test datasets used in three experiments. Each row indicates the specific experiment, dataset, and the comparison group, healthy controls (HC), episodic migraine (EM), and chronic migraine (CM) patients

**Question #3.**

Reformulate the sentence related to Freesurfer's recon-all. The message is incorrect. The recon-all allows obtaining cortical surfaces. By applying the Desikan atlas, it is possible to obtain volume, area, and thickness measurements for cortical regions.

**Response:** We have revised the sentence related to Freesurfer's recon-all to accurately reflect its functionality. The corrected sentence now reads:

"The recon-all pipeline in FreeSurfer allows for the reconstruction of cortical surfaces. By applying the Desikan-Killiany atlas, we obtained measurements of volume, area, and thickness for cortical regions."

**Question #4.**

Check the acronyms. For example, at the beginning of the results, acronyms already used that had not been defined such as EM and CM are reported.

**Response:** We have reviewed the entire manuscript to ensure that all acronyms are defined upon their first use. The acronyms EM (Episodic Migraine) and CM (Chronic Migraine) are now clearly defined at their first mention in the results section.

**Question #5.**

There are several sentences in the results that should go in the discussion. E.g., page 18: ‘These significant enhancements … improvements across all evaluated performance metrics’.

**Response:** We have identified several sentences in the results section that are more appropriate for the discussion section. Specifically, the following sentences have been moved:

These significant enhancements affirm the healthy core's utility in fostering the generalization of classification models. By leveraging a harmonized subset of HCs from different datasets, our approach not only improves model accuracy but also ensures the models are robust and applicable across varying clinical and research settings. Thus, the healthy core concept represents a significant advancement in the development of machine learning classifiers for migraine detection, as evidenced by the substantial improvements across all evaluated performance metrics.

**Question #6.**

Modify the title of the paragraph '3.4. Discussions on Healthy Core'. It cannot be defined as a 'discussion'. It seems more like an in-depth analysis. I emphasize that this part of the result is not reported in the method section.

**Response:** The title of the paragraph '3.4. Discussions on Healthy Core' has been changed to '3.4. In-Depth Analysis of Healthy Core'. Additionally, we have ensured that this part of the analysis is now adequately reported in the methods section to maintain coherence and clarity. The methods section now includes a detailed description of the statistical and computational techniques used for the in-depth analysis of the healthy core, ensuring that readers can follow the methodology and understand its implementation and significance.
